# XChrom: A Cross‐Cell Chromatin Accessibility Prediction Model Integrating Genomic Sequence and Cellular Context

**DOI:** 10.1002/advs.78456

**Published:** 2026-10-28

**Authors:** Yuanyuan Miao, Xuan Liang, Wenwen Zhang, Dongmei Han, Yurun Li, Zhen Wang

**Affiliations:** ^1^ Shanghai Institute of Nutrition and Health Chinese Academy of Sciences University of Chinese Academy of Sciences Shanghai China; ^2^ Zhejiang Key Laboratory of Signaling Mechanisms in Tumor Progression and Intervention School of Life Science Hangzhou Institute for Advanced Study University of Chinese Academy of Sciences Hangzhou China; ^3^ Yuhang Institute of Medical Science Innovation and Transformation Hangzhou China

**Keywords:** chromatin accessibility, deep learning, multimodal integration, regulatory genomics, single‐cell sequencing

## Abstract

Single‐cell chromatin accessibility offers unique insights into transcriptional regulation beyond gene expression. However, paired datasets of these two modalities remain relatively scarce, and existing computational models cannot simultaneously predict chromatin accessibility for unseen genomic regions and cells. Here, we present XChrom, a deep learning framework for genome‐wide cross‐cell chromatin accessibility prediction that integrates genomic sequence and single‐cell transcriptomics‐derived cell identity into a unified model. Comprehensive evaluations show that XChrom consistently outperforms existing methods in cross‐region and cross‐cell predictions, and uniquely enables cross‐both predictions. It also supports cross‐sample prediction when batch effects are properly corrected. Cross‐species analyses demonstrate XChrom's robust performance and its ability to capture evolutionarily conserved regulatory rules. Applied to peripheral blood mononuclear cells from COVID‐19 patients, XChrom accurately identifies transcription factor activities in novel but related cell states and reveals pathogenesis‐associated regulatory alterations. XChrom is available as an open‐source Python package, facilitating single‐cell transcriptional regulation research.

## Graphical abstract

XChrom is a deep learning framework that integrates genomic sequence and single‐cell context to predict chromatin accessibility. It generalizes to unseen cells and genomic regions, enabling cross‐sample and cross‐species analysis. Applied to COVID‐19 patient data, XChrom reveals key transcriptional regulatory mechanisms and disease‐associated alterations, offering a robust tool for single‐cell multi‐omics research.

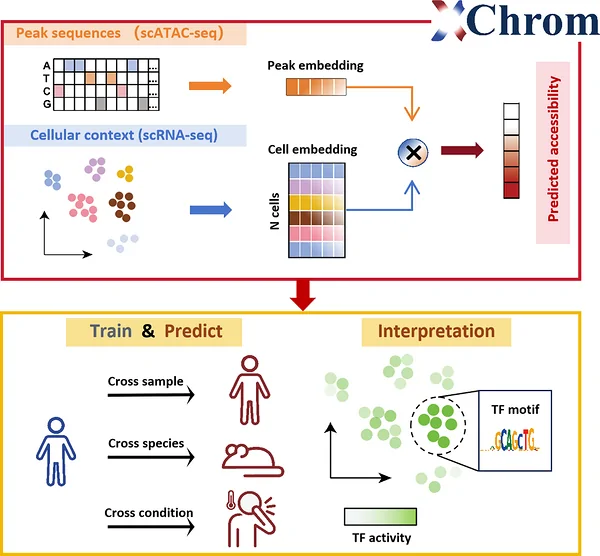

## 1 Introduction

The rapid advancement of single‐cell sequencing technologies has greatly improved our ability to dissect cellular heterogeneity at single‐cell resolution. For instance, single‐cell RNA sequencing (scRNA‐seq) has been widely used to identify novel cell subpopulations and reveal the diversity of cellular identities and functions. Nevertheless, despite its strengths, scRNA‐seq alone remains insufficient to uncover the transcriptional regulatory mechanisms underlying gene expression programs [1]. To address this gap, it is essential to consider chromatin accessibility, which is a key layer of epigenetic regulation and provides critical insights into gene regulatory dynamics. In this context, single‐cell assay for transposase‐accessible chromatin with sequencing (scATAC‐seq) profiles genome‐wide open chromatin regions at single‐cell resolution, offering a complementary view of regulatory heterogeneity and cell state transitions [2].

Although experimental techniques for simultaneously profiling RNA and ATAC modalities in a single cell have become feasible [3] (e.g., 10x Multiome), compared with scRNA‐seq, such multimodal datasets remain relatively limited due to the constraint of experimental costs. Several computational approaches have been developed to generate scATAC‐seq profiles [4], which can be broadly categorized into two approaches. The first class of methods leverages established mappings between RNA and ATAC modalities. For instance, Seurat [5] projects unimodal scRNA‐seq data into a multimodal embedding space derived from multi‐omics references, identifies *k*‐nearest neighbors (KNNs), and predicts chromatin accessibility by transferring ATAC profiles from the neighbors. Methods such as LS_Lab [6] and MultiVI [7] instead learn a shared latent space from multi‐omics reference data and train a joint representation model to reconstruct each modality, thereby enabling decoding from RNA to ATAC signals. Although these approaches have shown success in cell embeddings and data imputation, a key limitation is that they fail to exploit the underlying DNA sequence information of open chromatin regions. As a result, they are insufficient to capture sequence‐driven transcriptional regulatory relationships. It is well established that lineage‐specific transcriptional regulation is primarily determined by regulatory DNA sequences [8]. Furthermore, these models are constrained to predicting chromatin accessibility only within the predefined genomic regions included in the training data, which limits their applicability for predicting entirely novel sequences.

The second class of methods focuses on sequence‐based modeling. scBasset [9] represents a notable example, employing a multi‐task convolutional neural network (CNN) to predict binary chromatin accessibility across multiple cells. scBasset adopts the architectures of sequence‐to‐function models used for bulk sequencing data, which have been shown to be effective in elucidating the effects of sequence variations and uncovering regulatory rules [10]. Although scBasset captures *cis*‐regulatory features encoded in DNA sequences and performs well in cell clustering and data denoising, it cannot generalize to unseen cells, limiting its practical applicability to new samples or cell states.

To overcome these limitations, we developed XChrom, a multimodal deep learning framework for predicting chromatin accessibility from sequences across single cells. It builds upon the CNN architecture of scBasset [9] to model DNA sequences, effectively capturing the regulatory information encoded in DNA. Importantly, it incorporates cell identity information as a key innovation, represented as low‐dimensional embeddings derived from scRNA‐seq data, into the model input. Our motivation stems from the fact that RNA‐derived features, such as the expression levels of key genes [11, 12] or binding status of core transcription factors (TFs) [13, 14], can serve as proxies for tissue or cell‐line identity, thereby enabling cross‐context generalization. We extend this concept to the single‐cell level, leveraging the abundance of scRNA‐seq data across diverse biological and disease contexts. This framework enables predicting chromatin accessibility for arbitrary genomic sequences in cells that are related but not identical to those in the training set. Comprehensive benchmarking demonstrates that XChrom achieves excellent performance in both within‐sample and cross‐sample prediction scenarios. More importantly, through interpretability analyses, XChrom can reveal *cis*‐ and *trans*‐regulatory mechanisms across varying cellular contexts. In cross‐species prediction, it exhibits exceptional generalization by capturing evolutionarily conserved regulatory rules. For peripheral blood mononuclear cells (PBMCs) from COVID‐19 patients, XChrom accurately captures TF activities of a cell state that is rare in the training set and reveals dynamic TF activities associated with disease progression. Together, these results establish XChrom as a robust and generalizable framework, enabling exploration of epigenetic regulation in development and disease directly from transcriptomic data. XChrom is implemented as an open‐source Python package, with detailed tutorials freely available at https://xchrom.readthedocs.io/en/latest/.

## 2 Results

### 2.1 Overview of XChrom

XChrom is a deep learning model that integrates sequence information and cell identity to predict chromatin accessibility probabilities for each genomic region across all cells. It is trained on paired scATAC‐seq and scRNA‐seq data from the same cells. The core design is based on the premise that the accessibility potential of genomic regions is determined by both sequence content and cellular context. Specifically, XChrom is structured as a hybrid neural network (Figure 1a), with the first component being a sequence encoding module using a CNN. This module takes a 1344‐bp sequence centered around each peak from scATAC‐seq data as input. After one‐hot encoding, the sequence is processed through seven convolutional blocks, each containing a one‐dimensional convolutional layer, a batch normalization layer, a max pooling layer, and a Gaussian error linear unit (GELU) activation function. Finally, an additional convolutional layer and a fully connected layer convert each input sequence into a 32‐dimensional peak embedding. The second component is a cell identity encoding module. As input, XChrom receives cell embeddings computed from scRNA‐seq data of the paired dataset (e.g., embeddings from principal component analysis (PCA)). Following *Z*‐score normalization and layer normalization, the initial cell embedding matrix is transformed through two fully connected layers, producing a final 32‐dimensional cell embedding matrix. The peak embedding and cell embedding matrix are integrated via matrix multiplication, generating predicted chromatin accessibility probabilities for a given genomic sequence across all cells (Figure 1a). The model is trained using the standard binary cross‐entropy loss function and optimized using the Adam optimizer (Methods).

**FIGURE 1:**
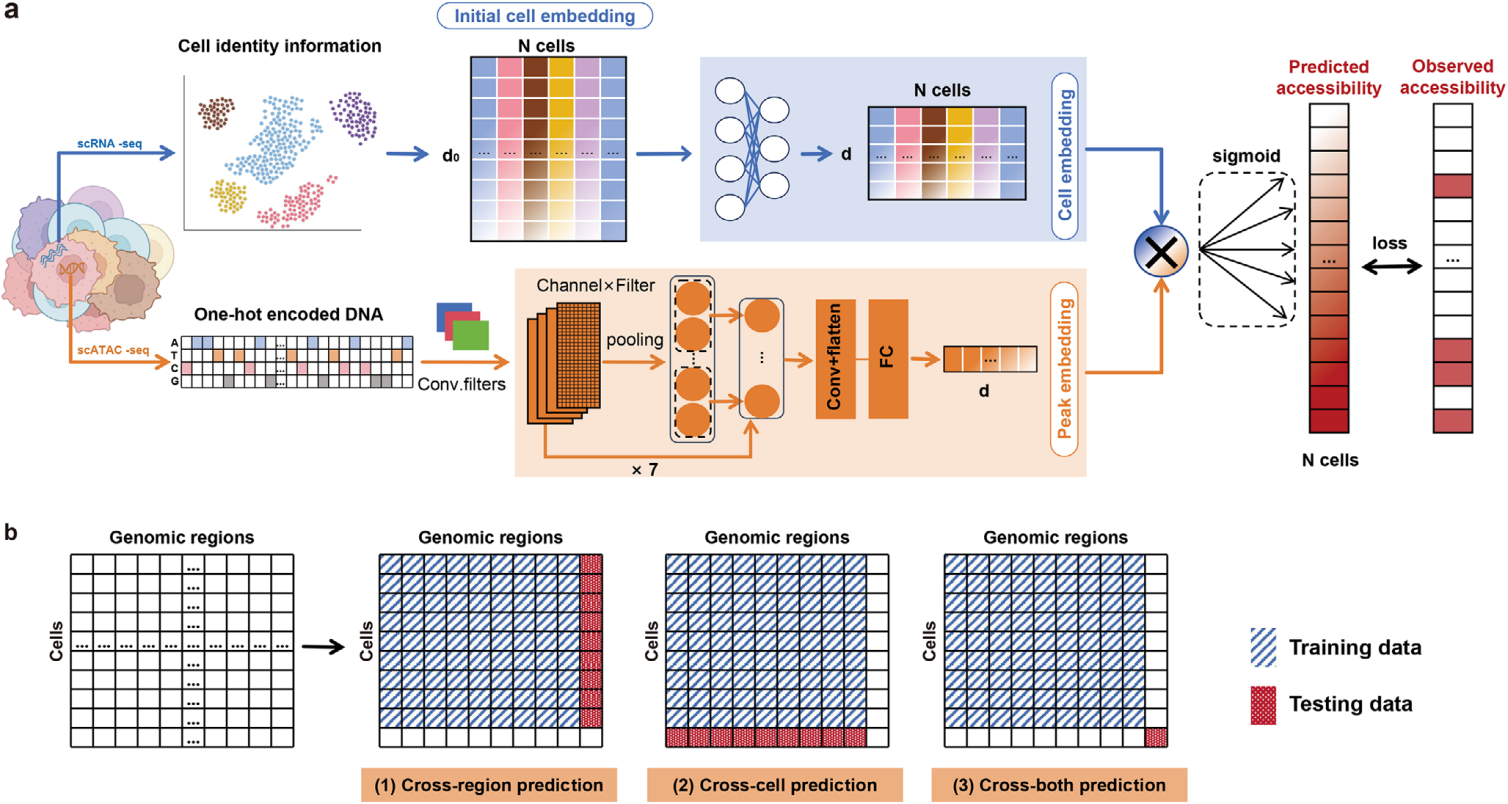
XChrom architecture and test tasks. (a) XChrom is a hybrid neural network with two distinct inputs. The first input is the one‐hot encoding of peak sequences (1344 bp) from scATAC‐seq data, processed through multi‐layer convolutions to ultimately generate a peak embedding for each sequence. The second input is an initial cell embedding matrix extracted from paired scRNA‐seq data, which is then transformed through dense layers to yield the final cell embedding matrix. These two sets of embeddings are integrated using matrix multiplication to predict the chromatin accessibility probabilities of a given sequence across all cells. “Conv” refers to convolutional layers, and “FC” refers to fully connected layers. *d* represents the dimensionality of the final peak and cell embeddings (default 32), while *d*_0_ represents the dimensionality of the initial cell embedding. The cellular illustrations were created with BioRender.com. (b) The three test tasks for XChrom: the cross‐region prediction, testing new regions not included in the training set; the cross‐cell prediction, testing new cells not included in the training set; and the cross‐both prediction, testing both unseen cells and unseen regions.

To comprehensively assess the predictive performance of XChrom, we divided the test set into three parts (Figure 1b): (i) the “cross‐region” test set, which evaluates the model's ability to predict new genomic regions not seen during training; (ii) the “cross‐cell” test set, which assesses the model's generalization to new cells not included in the training set; and (iii) the “cross‐both” test set, which tests prediction of unseen genomic regions and unseen cells, representing the most challenging task. For each test, we used two binary classification metrics‐the area under the receiver operating characteristic curve (auROC) and the area under the precision‐recall curve (auPRC)‐as primary evaluation metrics, which were summarized at the overall, per‐cell, and per‐peak levels (Methods).

### 2.2 XChrom Accurately Predicts Chromatin Accessibility Across Three Test Tasks

We collected five publicly available paired scRNA‐seq and scATAC‐seq datasets for benchmarking against existing models, covering both human and mouse species (Figure 2a). Each dataset underwent consistent preprocessing, training/validation/test set division, and model training (Methods). The model converged after 1000 epochs of training across all datasets, as evidenced by validation set metrics (Figure S1). In the cross‐region prediction test, we compared XChrom with scBasset [9], a state‐of‐the‐art chromatin accessibility prediction model that relies solely on sequence information. Although performance varied across datasets, XChrom's performance trend was highly consistent with that of scBasset across all evaluation metrics, with non‐significant or only minor differences in overall, per‐cell, and per‐peak auROC/auPRC (Figure 2b and Figure S2; statistical tests are provided in Table S1). This indicates that XChrom, as a multimodal model, retains the strengths of sequence models in cross‐region prediction.

**FIGURE 2:**
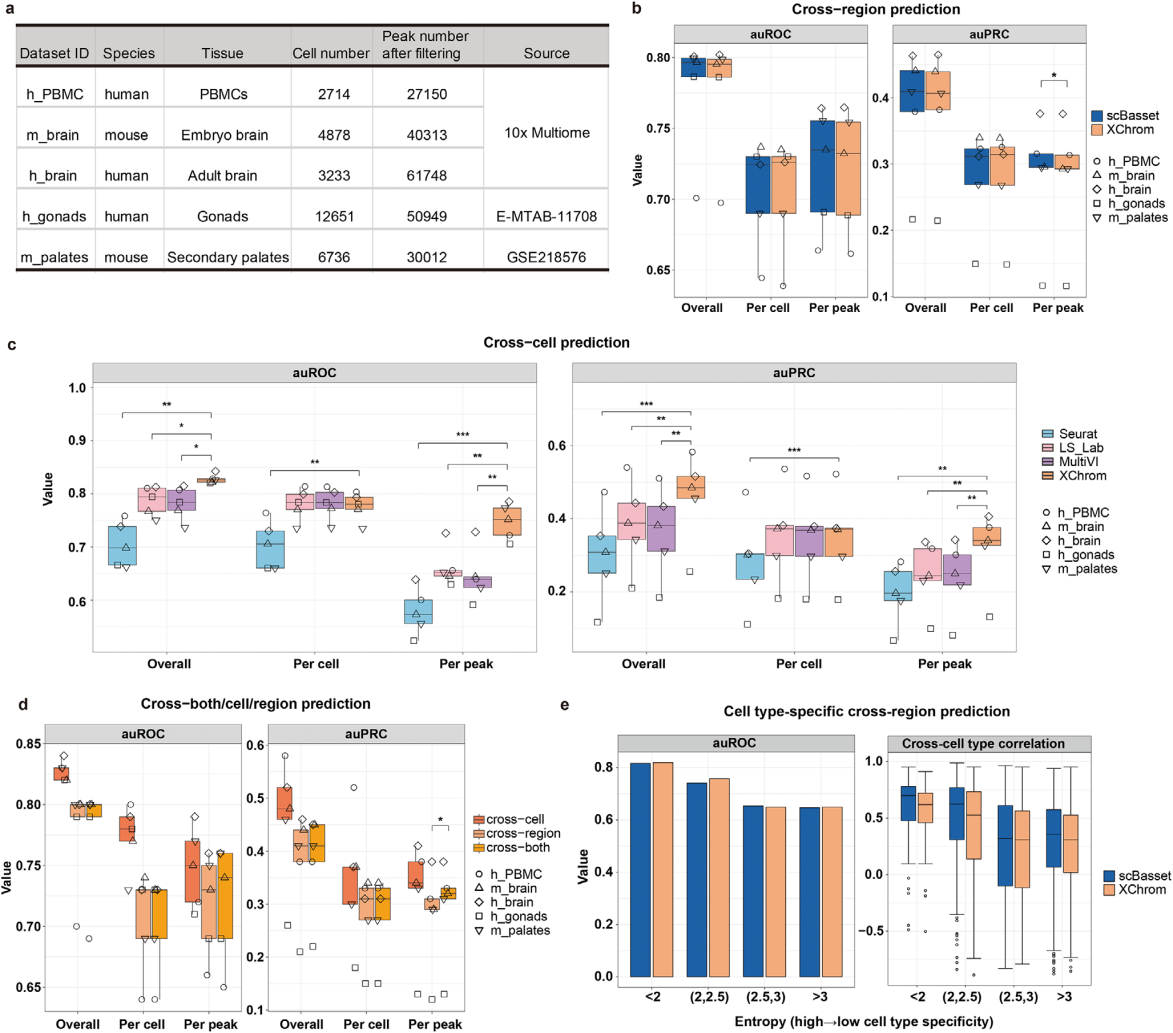
XChrom's classification performance and benchmarking against other methods across three test tasks. (a) Basic information on the publicly available paired scRNA‐seq and scATAC‐seq datasets used for benchmarking. (b) Comparison of prediction performance between XChrom and scBasset in the cross‐region prediction test. Statistical significance was assessed using two‐sided paired tests (Methods and Table S1). *N* = 5 datasets. Only significant comparisons are indicated: ^*^ *p* < 0.05; ^**^ *p* < 0.01; ^***^ *p* < 0.001. (c) Comparison of XChrom with Seurat, LS_Lab, and MultiVI in the cross‐cell prediction task. Statistical significance was assessed using two‐sided paired tests (Methods and Table S2). *N* = 5 datasets. Only significant comparisons are indicated: ^*^ *p* < 0.05; ^**^ *p* < 0.01; ^***^ *p* < 0.001. (d) Evaluation of XChrom's performance across three test tasks: cross‐both, cross‐region, and cross‐cell. Statistical significance was assessed using two‐sided paired tests (Methods and Table S3). *N* = 5 datasets. Only significant comparisons are indicated: ^*^ *p* < 0.05; ^**^ *p* < 0.01; ^***^ *p* < 0.001. (e) auROC and cross‐cell type correlation between model‐predicted and observed mean chromatin accessibility for XChrom and scBasset across entropy‐stratified peaks. The analysis was performed on 2714 held‐out test peaks from the cross‐region prediction task (entropy < 2, *n* = 58; 2< entropy < 2.5, *n* = 274; 2.5< entropy < 3, *n* = 639; entropy > 3, *n* = 1743).

Then, we evaluated the computational cost of XChrom and scBasset during training on the h_PBMC dataset and a larger PBMC10k dataset. Under the same 1000‐epoch setting using an NVIDIA GeForce RTX 3090 GPU, XChrom and scBasset showed broadly comparable training times and peak GPU memory usage. However, XChrom required more CPU memory, which is consistent with the additional use of scRNA‐seq‐derived cell state information (Figure S3).

In the cross‐cell prediction test, we compared XChrom with three single‐cell cross‐modal prediction models: Seurat [5], LS_Lab [6], and MultiVI [7] (Methods). These models were top‐performing in a previous benchmarking study [4]. XChrom significantly outperformed all other models in overall and per‐peak auROC/auPRC (Figure 2c; statistical tests are provided in Table S2). For per‐cell auROC/auPRC, XChrom performed comparably to LS_Lab and MultiVI, but significantly outperformed Seurat. The advantage of XChrom in per‐peak metrics indicates that it is more capable of accurately capturing cell‐specific information when predicting chromatin accessibility in unseen cells.

Finally, we evaluated XChrom's performance in the cross‐both prediction test, a task infeasible for all the other models. Figure 2d shows that, although this setting is more challenging, XChrom still achieves satisfactory prediction performance. For most metrics, its performance was not significantly different from that in the cross‐region and cross‐cell tasks (statistical tests are provided in Table S3). These results indicate that XChrom retains robust generalization ability when faced with unseen genomic regions and cells, demonstrating that it learns the general principle by which DNA sequences determine chromatin accessibility in a cell‐specific manner.

We performed additional analyses on the h_PBMC dataset by stratifying peaks according to their cell‐type specificity based on Shannon entropy (Figure S4a) and examining predicted accessibility patterns of held‐out peaks across cell types. Since peaks in different entropy groups have distinct auPRC baselines (Figure S4b), we relied on auROC for comparison. Both XChrom and scBasset showed higher auROC for highly specific peaks (entropy < 2) than for broadly accessible peaks (entropy > 3; Figure 2e), consistent with their stronger discrimination between accessible and inaccessible states for the former (Figure S4c). Likewise, the predicted accessibility profiles agreed better with the observed accessibility patterns across cell types for cell‐specific peaks (Figure 2e). Examination of the loss during training revealed that, in contrast to cell type‐specific peaks, broadly accessible peaks exhibited higher average loss, which mainly stemmed from poorer discrimination of negative states (Figure S4d). These results demonstrate that XChrom achieves better prediction performance for cell type‐specific peaks. Furthermore, the majority of highly specific peaks (entropy < 2) exhibited positive correlations between XChrom‐predicted accessibility and the expression of nearby genes across cell types (Figure S4e,f), indicating XChrom recapitulates their associated biological variations.

### 2.3 Chromatin Accessibility Data Predicted by XChrom have High Cell State Fidelity

Due to the inherent sparsity of scATAC‐seq, binarized data often contain a large number of false‐negative signals. Previous studies proposed that imputed scATAC‐seq data from a machine learning model can serve to remove such noise [9, 15, 16]. Therefore, we further examined XChrom's denoising capability on raw scATAC‐seq data by assessing the preservation of cell identity, which we defined as cell state fidelity. It was quantified using two metrics introduced by scBasset [9]: the neighbor score (NS), which quantifies cross‐modality neighborhood concordance by comparing scATAC‐seq and scRNA‐seq data, and the label score (LS), which measures the consistency of cell‐type labels within local neighborhoods (Methods). Neither the NS nor LS metric depends on the choice of clustering resolution for imputed scATAC‐seq data, though both rely on a parameter *k*, the number of nearest neighbors in the KNN graph. For datasets lacking prior cell type annotations, we used cluster indices identified via Leiden clustering of scRNA‐seq data as proxy labels for LS calculation. As expected, the NS and LS derived from denoised results showed high consistency with those from final cell embeddings, providing another perspective on monitoring convergence during model training (Figure S5a,b, Methods). Briefly, although XChrom usually converges on conventional accuracy metrics (loss, auROC, and auPRC) at relatively early training epochs (Figure S1), NS and LS, together with cell clustering performance, continue to be optimized at later epochs (Figure S5c,d).

First, we applied the trained model for each dataset to denoise cells in the whole dataset and compared the results among XChrom, scBasset, and raw scATAC‐seq data. Uniform Manifold Approximation and Projection (UMAP) visualizations revealed that the denoised data generally showed clearer separation among cell types in h_PBMC (Figure 3a) and the other four datasets (Figure S6). In particular, across multiple neighborhood resolution values of *k*, XChrom's denoised data consistently achieved higher NS and LS than both scBasset and raw scATAC‐seq data (Figure 3b; statistical tests are provided in Table S4). This trend was also supported by conventional clustering metrics, including the adjusted Rand index (ARI), the adjusted mutual information (AMI), and the average silhouette width (ASW) (Figure 3b; statistical tests are provided in Table S4). We further compared XChrom with two dedicated scATAC‐seq denoising/imputation methods, SCALE [15] and scCASE [17]. Across the evaluated cell‐state fidelity metrics, XChrom‐denoised accessibility profiles showed performance that was comparable to, and in several cases higher than, these methods (Figure S7; statistical tests are provided in Table S4). The strong preservation of cell‐type structure in XChrom‐denoised profiles may partly be attributed to XChrom's incorporation of paired scRNA‐seq‐derived cell embeddings (absent in scBasset, SCALE, and scCASE), which provides complementary information about cellular state when reconstructing chromatin‐accessibility profiles.

**FIGURE 3:**
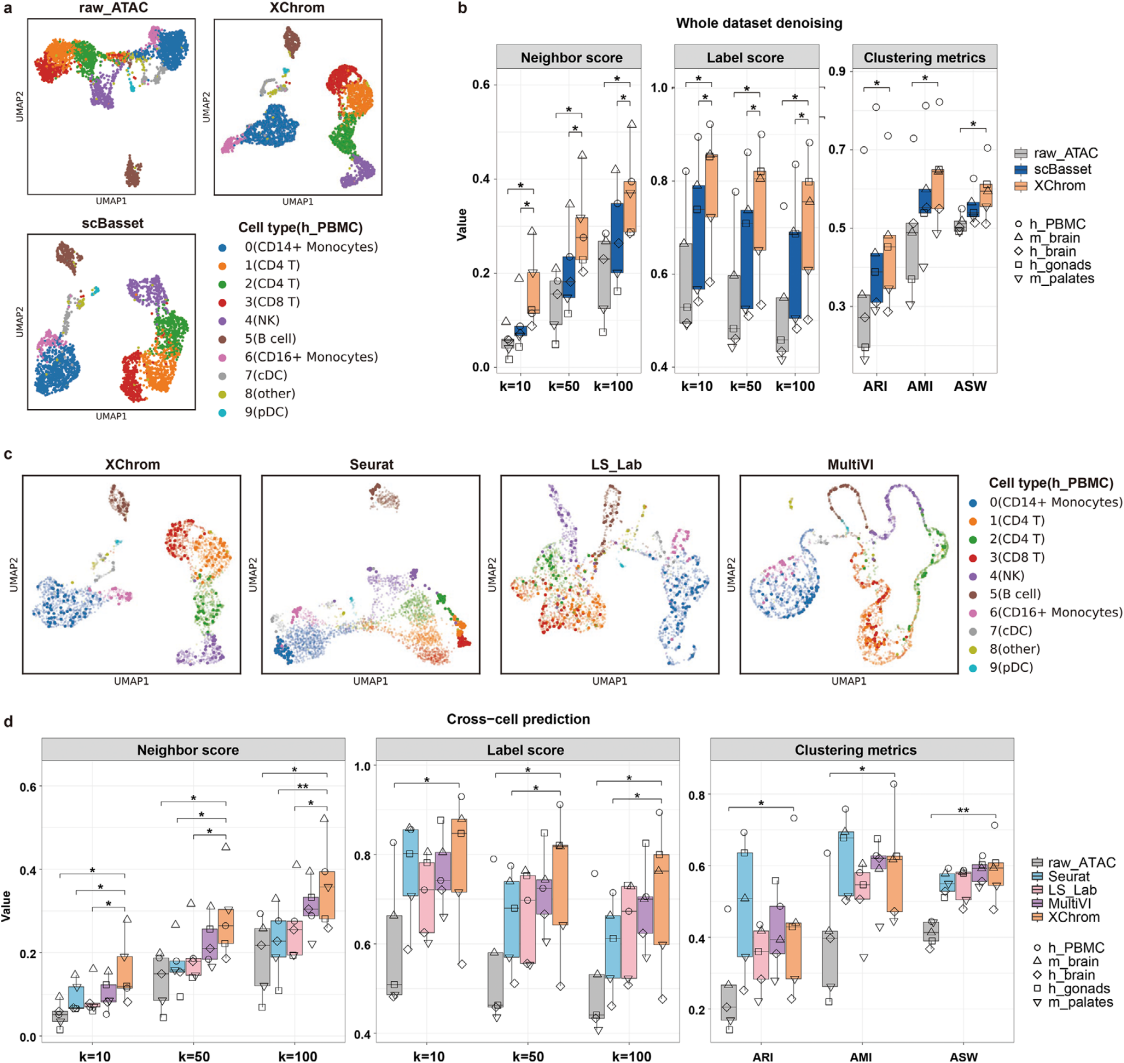
XChrom's prediction performance in cell state fidelity and benchmarking against other methods. (a) UMAP visualizations of the raw scATAC‐seq data (raw_ATAC), XChrom‐ and scBasset‐denoised results for the h_PBMC dataset, with cell type annotations for each cluster. (b) NS, LS, and clustering metrics for raw_ATAC, XChrom‐, and scBasset‐denoised results. NS and LS were calculated with *k* = 10, 50, and 100 neighbors. Statistical significance was assessed using two‐sided paired tests (Methods and Table S4). *N* = 5 datasets. Only significant comparisons are indicated: ^*^ *p* < 0.05; ^**^ *p* < 0.01; ^***^ *p* < 0.001. (c) UMAP visualizations of test cell predictions by Seurat, LS_Lab, MultiVI, and XChrom for the h_PBMC dataset. Larger points represent test cells, while background points represent training cells. (d) Evaluation of NS, LS and clustering metrics for test cell predictions, comparing XChrom with Seurat, LS_Lab, MultiVI, and raw_ATAC. Statistical significance was assessed using two‐sided paired tests (Methods and Table S2). *N* = 5 datasets. Only significant comparisons are indicated: ^*^ *p* < 0.05; ^**^ *p* < 0.01; ^***^ *p* < 0.001.

Subsequently, we evaluated XChrom with cross‐modal prediction models, including Seurat [5], LS_Lab [6], and MultiVI [7], with a specific focus on the cross‐cell test set for assessing cell state fidelity of unseen cells (Methods). XChrom consistently imputed correct cell labels for these test cells, as shown in UMAP visualizations (Figure 3c and Figure S8). When considering only the test cells, we found that XChrom and MultiVI performed the best in most cases (Figure 3d; statistical tests are provided in Table S2). However, in some instances, MultiVI's UMAP visualizations were less than ideal, showing a linear pattern and unclear boundaries between clusters (Figure 3c and Figure S8).

In conclusion, these results indicate that XChrom can not only effectively denoise existing scATAC‐seq data but also directly generate denoised results for cells lacking scATAC‐seq data when provided with cell identity information.

### 2.4 XChrom's Predictions Benefit From Key Model Components and Representative Cell Types

We conducted comprehensive ablation experiments on the h_PBMC dataset to dissect the contributions of model components to XChrom's predictions, including the cell module and sequence module. Under identical training/test splits and training durations (1000 epochs), we compared the default XChrom model against several distinct cell module variants: (i) **PCA_no_dense** (direct integration of 32‐dimensional paired scRNA‐seq PCA vectors without dense layers); (ii) **PCA_larger_dense** (increase the capacity of dense layers processing the PCA representation); (iii) **scVI** (replace PCA‐derived cell representation with scVI [18]‐derived cell embeddings); (iv) **cell_index** (replace paired scRNA‐seq‐derived cell information with non‐informative cell indices); and sequence module variants: (i) **seq_index** (replace DNA‐sequence input with non‐informative sequence indices); (ii) **simpler_CNN** (a simplified sequence encoder with three CNN layers); (iii) **Sei** (replace DNA‐sequence input with Sei [19]‐derived sequence features). The detailed model settings were described in Methods. We evaluated predictive accuracy across the cross‐region, cross‐cell, and cross‐both tasks, and computed the NS and LS for both training and held‐out test cells. Our analysis yielded some key results:

(1) The cell module is primarily responsible for generalizing to unseen cells.

**PCA_no_dense** substantially reduced predictive performance across the three tasks, particularly in overall and per‐cell auROC/auPRC (Figure 4a and Figure S9a). Interestingly, this variant showed disproportionately high NS values despite its lower prediction accuracy. This indicates that directly injecting the PCA representation, without dense layers, will cause the RNA‐derived cell‐state information to dominate the integrated representation, thereby preserving RNA neighborhood structure without necessarily improving sequence‐based chromatin‐accessibility prediction. In contrast, **PCA_larger_dense** did not result in a substantial improvement across the evaluated metrics (Figure 4a and Figure S9a). **scVI** produced highly similar predictive performance across the evaluated metrics to that of the default model (Figure 4a and Figure S9a). Replacing paired scRNA‐seq‐derived cell information with non‐informative cell indices (**cell_index**) had a much stronger effect. In particular, per‐cell predictive performance on the cross‐cell task was largely lost (Figure 4a and Figure S9a), and the inferred cell‐state structure of test cells was not correctly recovered (Figure S9b). Some per‐peak predictive performance remained, likely because sequence‐related and sequencing‐depth information could still contribute to prediction.

**FIGURE 4:**
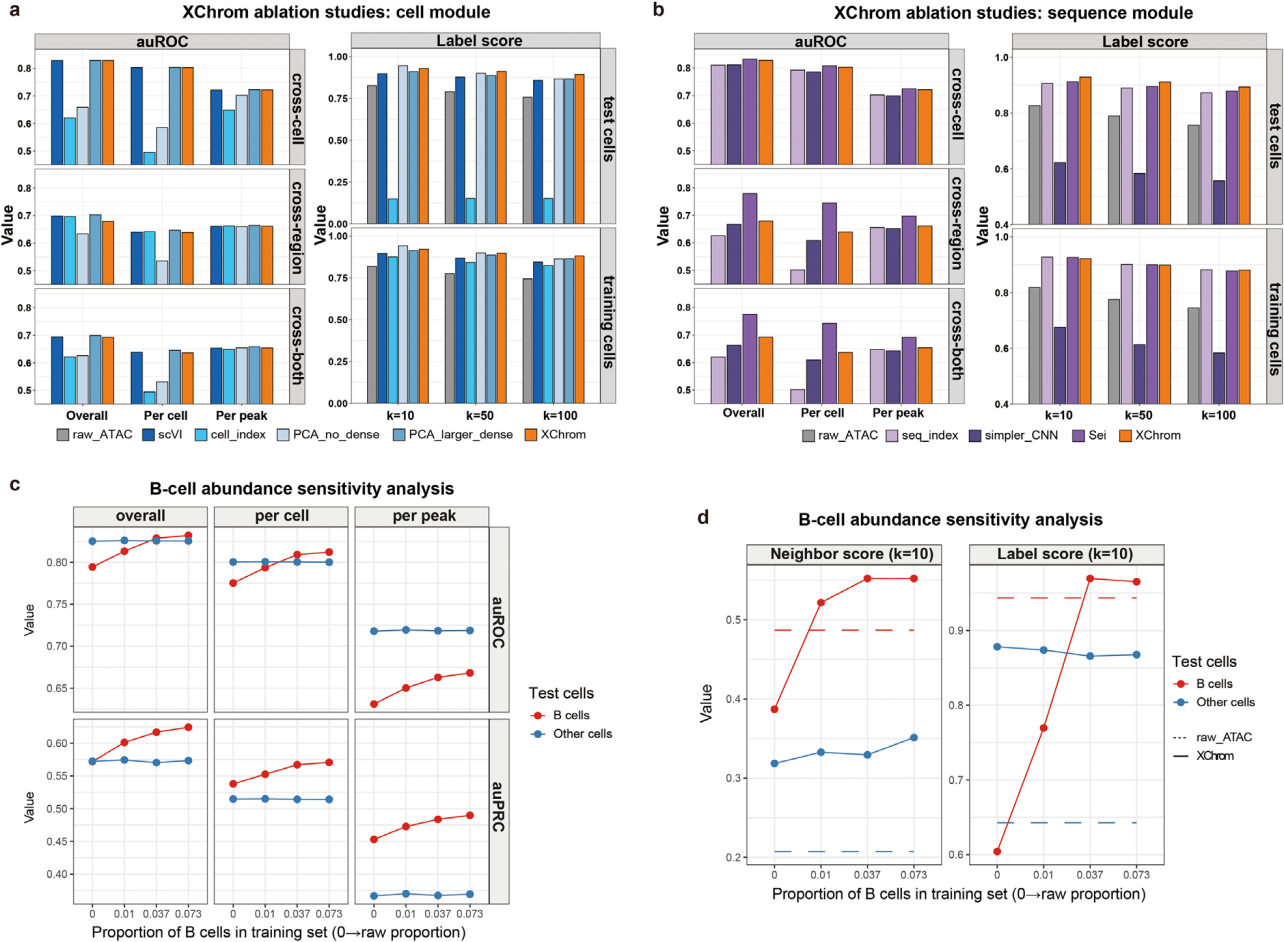
Evaluation of XChrom prediction sensitivity across varying model components and cell type abundances in the h_PBMC dataset. (a) Ablation study of the cell module. Model variants include: (i) **PCA_no_dense** (direct integration of 32‐dimensional paired scRNA‐seq PCA vectors with the sequence module without dense layers); (ii) **PCA_larger_dense** (replace the default cell‐processing network with a higher‐capacity neural network); (iii) **scVI** (use scVI‐derived cell embeddings instead of PCA‐derived embeddings); and (iv) **cell_index** (paired scRNA‐seq‐derived cell information was replaced with non‐informative cell indices). XChrom represents the default configuration. (b) Ablation study of the sequence module. Model variants include: (i) **seq_index** (DNA‐sequence input was replaced with non‐informative sequence indices and prediction relied only on the cell‐context branch); (ii) **simpler_CNN** (a simplified sequence encoder with three CNN layers); (iii) **Sei** (utilize Sei‐derived sequence features to replace the default sequence encoder). XChrom represents the default configuration. (c) Prediction performance of B cells and remaining cells, measured by auROC and auPRC, across the cross‐region, cross‐cell, and cross‐both settings for XChrom models trained with varying B‐cell proportions (0%, 1%, 3.7%, and 7.3% (original B‐cell proportion)) in the training set. (d) NS and LS (*k* = 10) of B cells and remaining cells calculated from denoised accessibility profiles of the test set generated by models trained with the indicated B‐cell proportions.

These indicate that an appropriate transformation of cell‐state representation is important, whereas increasing the complexity of this module or replacing PCA with another dimensionality‐reduction method provides little additional benefit.

(2) The sequence module is primarily responsible for generalizing to unseen genomic regions.

**Seq_index** resulted in a marked reduction in cross‐region prediction performance, demonstrating that DNA‐sequence information is essential for generalization to genomic regions. In contrast, NS and LS remained relatively well preserved (Figure 4b and Figure S9c), indicating that RNA‐derived cell‐state information can still be retained in the final predicted chromatin‐accessibility representation even in the absence of sequence input. **Simpler_CNN** showed reduced cross‐region performance and poorer NS/LS in both training and test cells compared with the default model (Figure 4b and Figure S9b,c). **Sei** substantially improved cross‐region prediction while maintaining cell‐state structure (Figure 4b and Figure S9c). These results indicate that the quality and capacity of the sequence representation directly influence the ability of XChrom to generalize to unseen genomic regions. Incorporation of larger pretrained regulatory sequence models can provide an advanced extension of the framework.

To assess XChrom's performance when a cell type is underrepresented or absent from the training data, we trained models on fixed‐size h_PBMC training sets containing different proportions of B cells (0%, 1%, 3.7%, and the original 7.3%) and evaluated them on the same test set containing both B cells and other cells (Methods).

For B cells in the test set, overall, per‐cell, and per‐peak auROC/auPRC progressively improved as the proportion of B cells in the training set increased, while predictive performance for the other cells remained largely unchanged (Figure 4c). Furthermore, the overlap of B cell‐specific peaks identified independently from the predicted test set and from the observed raw scATAC‐seq data increased progressively with the proportion of B cells included in the training set (Figure S9d). When B cells were completely absent from the training set, NS and LS calculated for predicted B cells were lower than those obtained from the raw ATAC data (Figure 4d), and B cells were poorly separated from other cell types in the UMAP representation (Figure S9e). As the proportion of B cells in the training set increased, both NS and LS progressively improved and exceeded the corresponding values calculated from the raw ATAC data (Figure 4d), consistent with the UMAP representation (Figure S9e).

Together, these results indicate that XChrom's generalization ability is limited when a cell type is completely absent from the training data. However, introducing even a small proportion of B cells into the training set improved prediction accuracy and reconstruction of B‐cell identity in the test set.

### 2.5 XChrom can Predict New Samples From the Same Tissue or Organ as the Training Set

The preceding study validated the model's performance within a single sample, where the scRNA‐seq data in both the training and test sets are inherently homogeneous, without batch effects. However, in most practical scenarios, the objective is to predict completely new and independent samples from a similar tissue or organ as the training set. To address this, we further evaluated XChrom's performance in a cross‐sample prediction scenario. We collected a dataset of paired scRNA‐seq and scATAC‐seq from bone marrow mononuclear cells (BMMCs) [20], which includes multiple batches arising from donor sources and sequencing platforms. A total of five samples were randomly selected as the training set, and five as the test set, employing a one‐to‐one cross‐sample training and testing approach. First, we evaluated the effect of different scRNA‐seq preprocessing and integration strategies on XChrom's cross‐sample prediction, including Scanpy ingest [21], Harmony [22], Scanorama [23], and scVI [18]. These approaches showed broadly comparable performance and consistently outperformed the analysis without batch correction (Figure S10). Based on these results, Harmony was used for the main cross‐sample analysis (Figure S11), and the resulting batch‐corrected cell embedding matrix was used as input to XChrom.

Second, we compared the performance of cross‐sample prediction to within‐sample prediction by XChrom. As shown in Figure 5a, cross‐sample prediction resulted in a significant decrease in overall, per‐cell, and per‐peak auROC (statistical tests are provided in Table S5). However, the 95% confidence intervals for the differences indicated that the magnitude of the reductions was limited. In the cell state fidelity assessment on the test set, the NS and LS values for cross‐sample prediction were lower than those observed in the within‐sample results, but XChrom still outperformed the raw scATAC data, particularly in terms of NS, where this superiority was statistically significant (Figure 5a; statistical tests are provided in Table S5). These findings suggest that batch effects between samples do influence XChrom's predictive performance, but XChrom remains applicable for predicting new samples if these batch effects are properly corrected.

**FIGURE 5:**
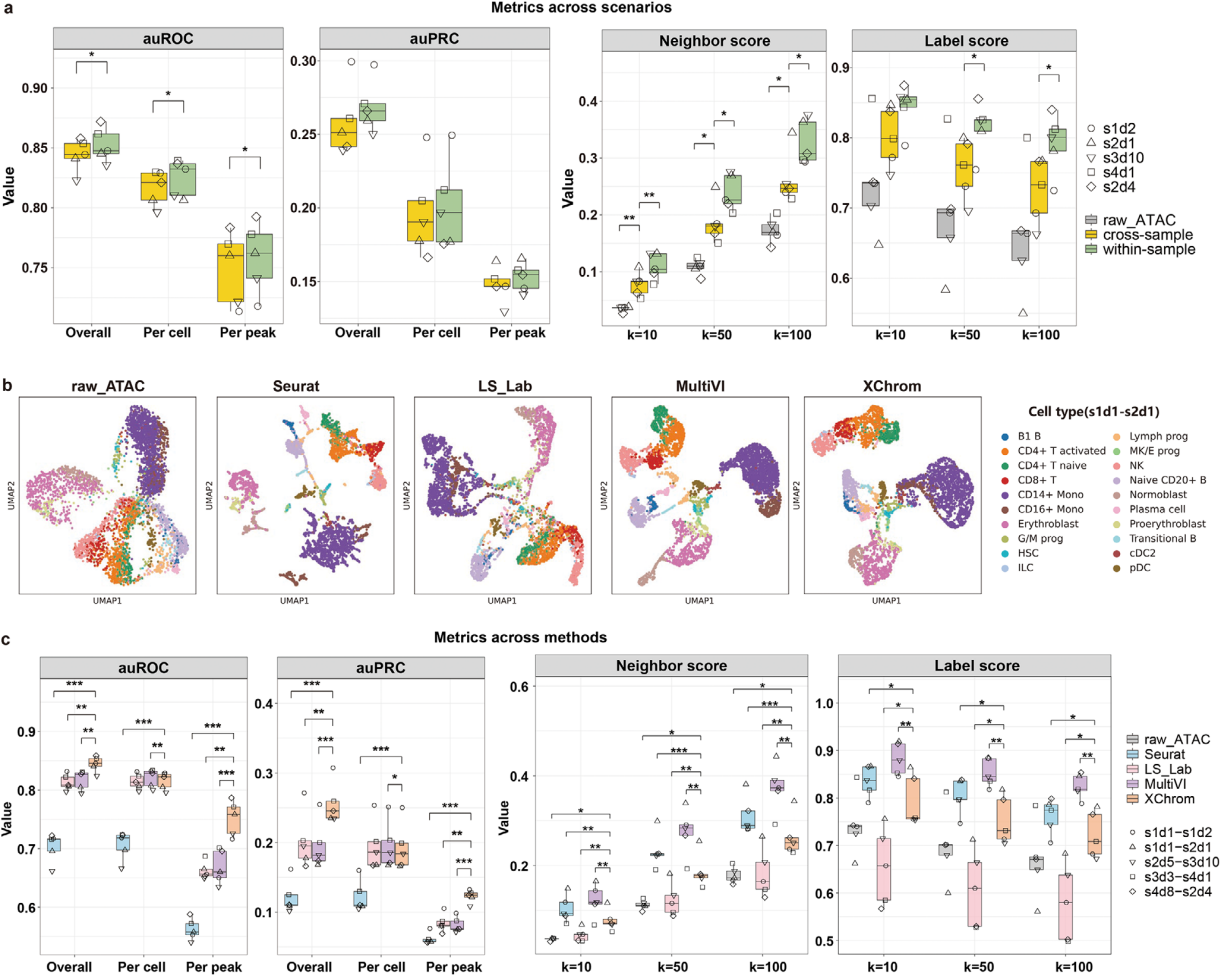
XChrom's prediction performance and benchmarking against other methods in a cross‐sample scenario. (a) Comparison of within‐sample and cross‐sample predictions by XChrom for classification performance and cell state fidelity. Only sample names of the test sets are labeled. Statistical significance was assessed using two‐sided paired tests (Methods and Table S5). *N* = 5 samples. Only significant comparisons are indicated: ^*^ *p* < 0.05; ^**^ *p* < 0.01; ^***^ *p* < 0.001. (b) Sample s1d1 from the BMMC dataset serving as the training set, with s2d1 as the test set. UMAP visualizations of raw scATAC‐seq data (raw_ATAC) of s2d1 and predicted data of s2d1 by Seurat, LS_Lab, MultiVI, and XChrom. (c) Comparison of raw_ATAC and cross‐sample predictions by Seurat, LS_Lab, MultiVI, and XChrom for classification performance and cell state fidelity. In the legend, sample names of ‘training set‐test set’ are labelled. Statistical significance was assessed using two‐sided paired tests (Methods and Table S6). *N* = 5 samples. Only significant comparisons are indicated: ^*^ *p* < 0.05; ^**^ *p* < 0.01; ^***^ *p* < 0.001.

Furthermore, we compared XChrom with Seurat [5], LS_Lab [6], and MultiVI [7] in the cross‐sample scenario (Figure 5b and Figure S12). The results demonstrated that XChrom significantly outperformed the other models across most classification metrics (Figure 5c; statistical tests are provided in Table S6). In terms of cell state fidelity, XChrom's performance was at a median level compared to other models, but outperformed raw scATAC‐seq data (Figure 5c). We hypothesize that, although batch effects were corrected during preprocessing using Harmony, residual batch differences between the training and test data may slightly impair XChrom's ability to preserve cell identity. Nonetheless, the results indicate that XChrom is adequate for predicting new samples from tissues or organs similar to those in the training set.

### 2.6 XChrom Enables Cross‐Species Prediction by Capturing Evolutionarily Conserved Regulatory Rules

Recent studies have demonstrated that sequence models trained on mouse scATAC‐seq data can accurately predict chromatin accessibility maps for other vertebrate genomes. These models have also revealed conserved regulatory logic and lineage‐specific gene regulation across vertebrates [24]. Such findings support Arendt's hypothesis that “cell identity across species is conserved and defined by a core gene regulatory network (GRN)” [25]. However, previous cross‐species models were limited to cells from the training set. Building on XChrom's unique cross‐both prediction capacity, we examined its potential for cross‐species prediction with varying genomic sequences and cellular contexts.

We collected and preprocessed paired scRNA‐seq and scATAC‐seq data from the primary motor cortex of four species: human, mouse, macaque, and marmoset, with each species containing at least three samples [26]. All scRNA‐seq data underwent uniform batch correction prior to training and prediction (Methods, Figure S13). Models were trained using a single sample from human (human_model: m1d1n) and mouse (mouse_model: mop3c2), respectively. We then assessed the cross‐species prediction performance of these two models on all remaining samples.

The results indicate that the predicted data from either the human_model (Figure 6a) or mouse_model (Figure S14a) could distinguish different cell types across each species. When applied to human test sets, the human_model and mouse_model performed comparably on most metrics, including per‐peak auROC/auPRC, per‐cell auROC (Figure 6b and Figure S14b) and NS/LS (Figure 6c and Figure S14c; statistical tests are provided in Table S7). However, when applied to mouse test sets, the mouse_model significantly outperformed both the human_model and the corresponding raw scATAC‐seq data across all metrics (Figure 6b,c and Figure S14b,c; statistical tests are provided in Table S7). This could result from the fact that, as a model organism, mouse samples were more homologous with minimal potential batch effects, rather than inherent species differences. When applied to macaque and marmoset test sets, human_model and mouse_model were broadly comparable. On several metrics‐including overall, per‐cell, and per‐peak auROC/auPRC‐the mouse_model even outperformed the human_model (Figure 6b and Figure S14b; statistical tests are provided in Table S7). Taken together, these results suggest that XChrom effectively captured evolutionarily conserved regulatory features and principles, demonstrating robustness even across distantly related species such as primates and rodents.

**FIGURE 6:**
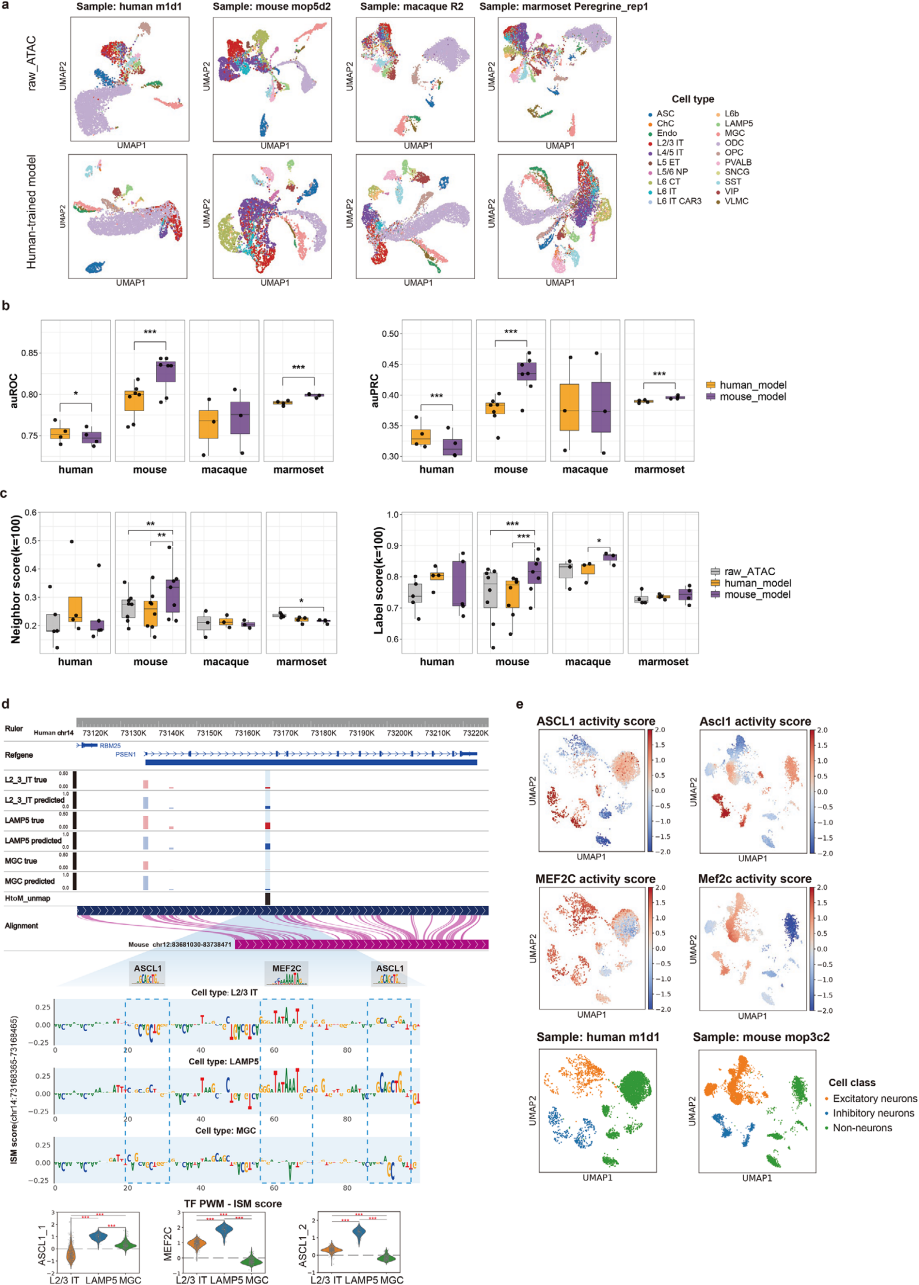
XChrom's prediction performance in a cross‐species scenario and its interpretability analysis. (a) UMAP visualizations of predictions generated by the human_model when applied to the test sets from four different species. Sample names are labelled in the plot. (b,c) Comparison of model performance between human_model and mouse_model using auROC/auPRC values (b), and NS/LS values (*k* = 100) (c), computed from predictions on all samples. Statistical significance was assessed using two‐sided paired tests (Methods and Table S7). For direct comparisons between the two models, sample sizes were *N* = 4 (human), *N* = 7 (mouse), *N* = 3 (macaque), and *N* = 4 (marmoset). Only significant comparisons are indicated: ^*^ *p* < 0.05; ^**^ *p* < 0.01; ^***^ *p* < 0.001. (d) Visualization of mouse_model predictions for a *PSEN1* regulatory sequence in the human sample (m1d1), with reference data from the WashU Comparative Epigenome Browser (https://epigenome.wustl.edu/BrainComparativeEpigenome/) [26]. The blue‐shaded sequence represents the human region (chr14: 73167855–73169199) that lacks homology to the mouse genome. Subsequences matching MEF2C and ASCL1 motifs are highlighted with blue dashed boxes in the cell‐type‐averaged ISM score plot. Below are per‐cell PWM‐ISM scores for MEF2C and ASCL1 in L2/3 IT (*n* = 335), LAMP5 (*n* = 78), and MGC (*n* = 211) cell types. The PWM‐ISM score is the dot product of the PWM and ISM scores at motif match sites (MEF2C at chr14:73168422‐73168436, ASCL1 at chr14:73168385‐73168397 and chr14:73168451‐73168463). A two‐sided Wilcoxon rank‐sum test was performed for statistical significance (Table S8); only significant comparisons are indicated: ^*^ *p* < 0.05; ^**^ *p* < 0.01; ^***^ *p* < 0.001. PWM: position weight matrix. (e) UMAP visualizations of MEF2C and ASCL1 motif activity predicted by the mouse_model across different cell types in the human sample (left) and the mouse sample (right).

A key advantage of sequence models is their interpretability [27], which can be utilized to further investigate the conservation of regulatory mechanisms. We calculated the TF motif activity at the single‐cell level, defined by the difference predicted by XChrom when the motif sequence was inserted into the background sequence [28] (Methods). We analyzed the cross‐species conservation of core TF activities across cell types (Methods). The average correlation of TF activity across cell types was high between the following pairs: 1) human_model predictions for both human and mouse samples (0.61); 2) mouse_model predictions for both human and mouse samples (0.67); 3) human_model predictions for the human sample and mouse_model predictions for the mouse sample (0.47, Figure S15). These results demonstrated that the conservation of cell type‐specific TF activity patterns was captured by XChrom.

To verify whether the model relies solely on sequence similarity for cross‐species prediction, we analyzed the neurodegenerative disease‐related gene *PSEN1* [29]. As shown in Figure 6d (representative cell types) and Figure S16a (other cell types), in the human sample (m1d1), a sequence that lacks homology to the mouse genome is accessible in the raw scATAC‐seq data. Despite not encountering this sequence during training, the mouse_model accurately predicted its accessibility. Further in silico saturation mutagenesis (ISM) was used to evaluate the importance of each site in this sequence [30] across cells in the human sample. Combined with FIMO [31] motif scanning results (Methods), we found that binding sites of MEF2C and ASCL1 exhibited higher regulatory importance in inhibitory neurons (e.g., LAMP5) compared with excitatory neurons (e.g., L2/3 IT) and non‐neurons (e.g., MGC), consistent with the higher real and predicted accessibility of this sequence in inhibitory neurons (Figure 6d and Figure S16a,b). These two TFs were also activated in inhibitory neurons in both human and mouse samples, as identified by XChrom's TF activity analysis (Figure 6e and Figure S16c,d). Previous research has shown that MEF2C plays a key role in early brain development, with mutations linked to various neuropsychiatric disorders [32, 33], while ASCL1 is a major regulator of neuronal fate determination during central nervous system development, particularly involved in generating inhibitory GABAergic interneurons [34, 35].

We also analyzed another gene, *DNAJC5* (also known as *CSPα*), which plays a crucial role in synaptic vesicle recycling and neurotransmitter release [36]. In the gene body, a human‐specific sequence was identified, with higher raw and predicted accessibility in excitatory neurons. Binding sites of both TEAD2 and GLI2 showed higher predicted importance in this sequence within these cell types, with their activation observed in both human and mouse samples (Figure S17). Previous studies have shown that TEAD2 participates in the mouse embryonic neural tube closure process [37], while GLI2 is closely related to neurogenesis, neuronal migration, and differentiation [38].

In summary, despite genomic differences between species, XChrom does not rely solely on sequence similarity for cross‐species predictions. Instead, it captures conserved transcriptional regulatory mechanisms, enabling robust transfer and generalization capabilities across species.

### 2.7 XChrom Predicts TF Activities of Novel Cell States and Regulatory Changes Across Different COVID‐19 Conditions

To explore the potential application of XChrom for new samples with different physiological and pathological conditions, we applied it to a single‐cell sequencing dataset of PBMCs from COVID‐19 patients, including severe, mild, and convalescent cases [39]. Although the dataset contains both scRNA‐seq and scATAC‐seq data, they are not paired. Therefore, we used a public paired scRNA‐seq and ATAC‐seq dataset from healthy donors (PBMC10k, 10x Multiome) to train an XChrom model, and then predicted the epigenomic features of COVID‐19 patients based on their scRNA‐seq data. The PBMC10k and COVID‐19 scRNA‐seq data were harmonized prior to model training and prediction (Figure S18a). The unpaired COVID‐19 scATAC‐seq data served as an independent resource for validating the model's findings.

First, we inferred the TF activity across different cell types (Methods). The results showed that XChrom could accurately identify well‐established cell type‐specific TF activities, such as CEBPB (monocytes), TCF7 (T cells), PAX5 (B cells), and RUNX3 (NK cells) (Figure S18b). As the pathogenesis of COVID‐19 is highly associated with monocytes [39], we focused on this cell type in the following analysis (Figure 7a). In the processed COVID‐19 scRNA‐seq data, a subcluster of monocytes (R1) was annotated as non‐classical monocytes (ncMono). Differential analysis of TF activity in R1 compared to other subclusters revealed significantly higher activity of several TFs (e.g., IRF family members, SPI1, STAT2; two‐sided Wilcoxon rank‐sum test, Table S9), which were also significantly enriched in the corresponding subcluster (C1) in the COVID‐19 scATAC‐seq data (Figure 7b and Table S10, Methods). These TFs are known to play key roles in myeloid development, antiviral response, and inflammation [40, 41, 42, 43, 44, 45, 46].

**FIGURE 7:**
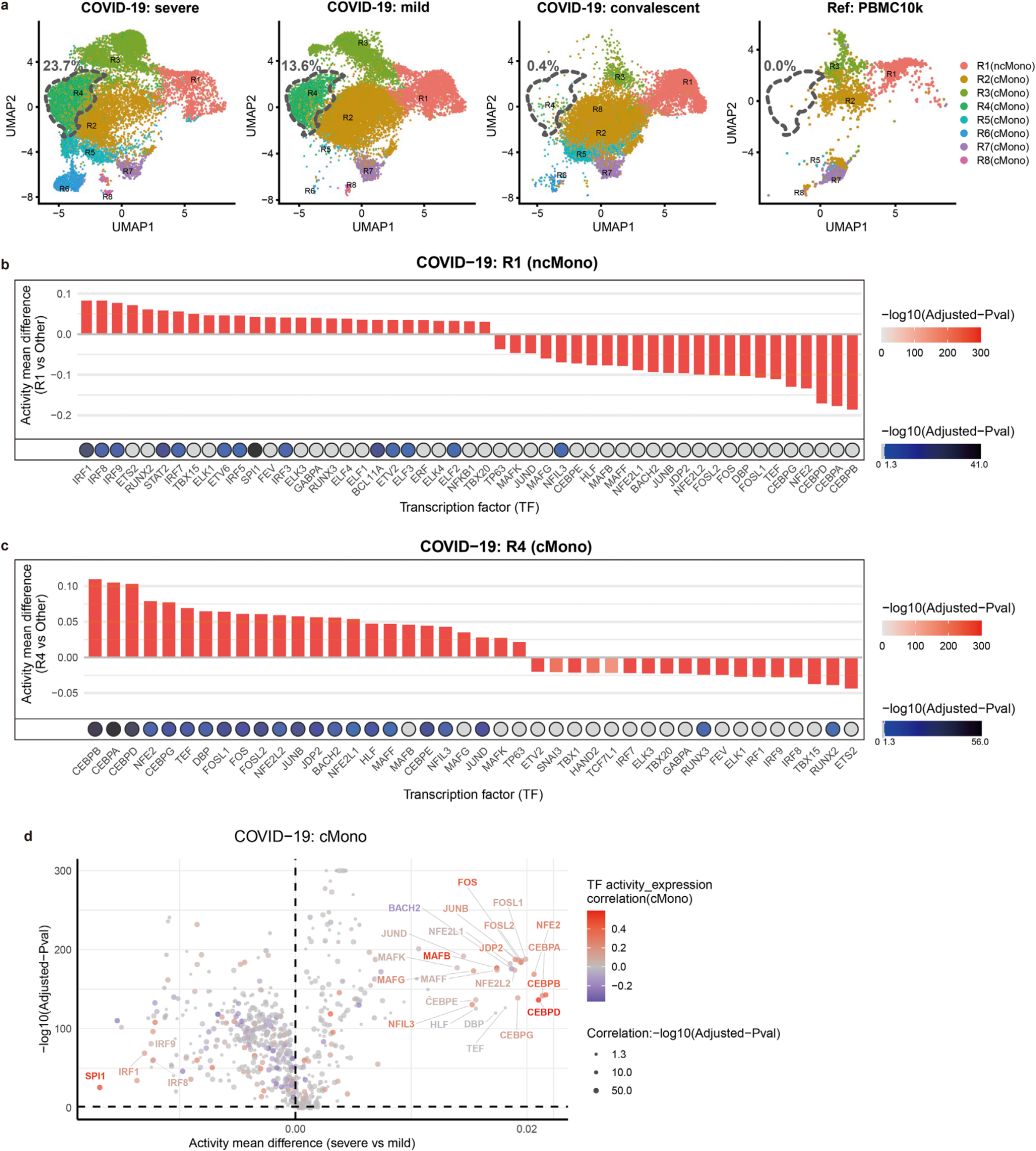
XChrom infers single‐cell TF activity and enables differential analysis across different COVID‐19 conditions. (a) UMAP visualizations of monocytes from the COVID‐19 scRNA‐seq dataset (including severe, mild, and convalescent patients) and the PBMC10k scRNA‐seq dataset. Subclusters of monocytes are labelled within each dataset. The grey dashed line indicates the R4 subcluster and its proportion. (b,c) Differential TF activity (bar plots) and motif enrichment (heatmaps) analysis in COVID‐19 monocyte subclusters. Colors reflect adjusted *p*‐values (two‐sided Wilcoxon rank‐sum test for TF activity; motif enrichment considered significant at ‐log10 (adjusted *p*‐value) > 1.3). R1 vs. other subclusters: bar plot shows the top 51 TFs with |mean difference| > 0.03 (positive values indicate higher activity in R1); heatmap shows motifs enriched in marker peaks of the corresponding subpopulation (C1) in the COVID‐19 scATAC‐seq data (b). R4 vs. other subclusters: bar plot shows the top 42 TFs with |mean difference| > 0.02 (positive values indicate higher activity in R4); heatmap shows motifs enriched in marker peaks of the corresponding subpopulation (C4) in the COVID‐19 scATAC‐seq data (c). (d) Differential analysis of TF activity in cMono between mild and severe COVID‐19 patients. The *x*‐axis represents the mean TF activity difference (severe‐mild), and the *y*‐axis represents the adjusted *p*‐value of the difference (two‐sided Wilcoxon rank‐sum test). The color of the dots indicates the Pearson correlation between TF activity and its expression, while the size of the dots indicates the adjusted *p*‐value of this correlation. The volcano plot uses a pseudo‐log transformation for axis scaling.

Next, we tested XChrom's generalization capability by predicting the epigenomic states of a novel classical monocyte (cMono) subpopulation (R4) that was rare in the training set (Figure 7a). In fact, R4 was annotated as HLA‐DR^lo^S100A^hi^ monocytes in the original study, which were mainly derived from hospitalized COVID‐19 patients (both mild and severe) but absent from convalescent cases [39]. TF activity prediction by XChrom and subsequent differential analysis on R4 revealed that C/EBP, FOS, and JUN family members were significantly more active in R4 than in other subclusters (two‐sided Wilcoxon rank‐sum test, Table S9). These TFs were also significantly enriched in the corresponding subpopulation (C4) in the COVID‐19 scATAC‐seq data (Figure 7c and Table S10), and this was consistent with the study showing that the dysfunctional HLA‐DR^lo^S100A^hi^ monocytes were characterized by CEBPD, CEBPE, FOSL2, FOSB, JUN, and NFIL3 in severe COVID‐19 patients [47]. These results indicate that XChrom can not only effectively recover the regulatory features of cell types contained in the training set, but also learn to extrapolate the regulatory logic to novel but related cell states.

Finally, we investigated regulatory changes during the progression of COVID‐19 by comparing the predicted TF activities in cMono between mild and severe COVID‐19 patients (Methods). We found that TFs such as C/EBP, FOS, JUN, and MAFB exhibited stronger activity in severe cases (two‐sided Wilcoxon rank‐sum test, Table S9), with their activity often positively correlating with gene expression (Figure 7d). These TFs are closely linked to the exacerbated inflammatory response and pro‐fibrotic processes observed in severe COVID‐19: activation of C/EBP TFs has been reported to promote profibrotic macrophage differentiation and trigger lung fibrosis in COVID‐19 [48]. FOSL1 has been shown to regulate the expression of pro‐inflammatory/anti‐inflammatory cytokines in macrophages and modulate pro‐fibrotic responses [49]. MAFB is a key driver in the transition from a macrophage‐to‐inflammatory‐fibrotic pathological state following SARS‐CoV‐2 infection, and its silencing significantly attenuates the associated pathogenic phenotype [50]. The balance between MAFB and MAF in lung macrophages is a critical determinant of COVID‐19 severity, with increased MAFB/MAF ratios weakening the type I interferon response, exacerbating inflammation, and enhancing fibrosis [51]. Additionally, MAFB can form heterodimers with JUN, FOS, and FOSL1/2 members of the AP‐1 family [52]. AP‐1, as a key downstream effector of the MAPK signaling pathway, is closely related to macrophage inflammatory responses induced by pathogen‐associated molecular patterns and is involved in the “cytokine storm” observed in viral infections [53, 54]. In contrast, the activities of IRF family members (IRF1/8/9) were significantly reduced in severe cases (two‐sided Wilcoxon rank‐sum test, Figure 7d and Table S9), which may impair the expression of interferons with antiviral roles. In fact, deficiency in type I interferon production and activity was observed in severe COVID‐19 patients [55], and a low‐interferon cMono state was predominant in severe cases [56]. Together, these results suggest that XChrom can make biologically plausible TF activity predictions across disease conditions.

## 3 Discussion

In this study, we present XChrom, a deep learning‐based framework capable of predicting genome‐wide chromatin accessibility at single‐cell resolution. The core innovation of XChrom lies in integrating DNA sequence information with cell identity derived from scRNA‐seq into a unified neural network architecture. Capturing the dependence between *cis*‐regulatory mechanisms and cellular context allows XChrom to overcome the limitations of existing methods, thereby enabling accurate inference of chromatin accessibility in both unseen sequences and unseen cells.

Through comprehensive evaluations, XChrom demonstrated superior predictive performance across various scenarios. Specifically, in cross‐region prediction within a sample, XChrom achieved comparable classification performance to scBasset [9] while showing better noise reduction capability. In cross‐cell prediction within a sample, XChrom outperformed Seurat [5], LS_Lab [6], and MultiVI [7] in classification performance, while maintaining the highest cell identity fidelity along with MultiVI. Additionally, XChrom was able to predict the accessibility probabilities of unseen genomic regions in unseen cells, a task that existing models fail to accomplish, although this capability requires related cell states to be properly represented in the training data. Our ablation studies further clarified the contributions of the two major model components. On one hand, the cell module was important for generalization to unseen cells, and prediction performance was relatively robust to the specific cell dimensionality‐reduction method. On the other hand, the sequence module was essential for generalization to unseen genomic regions, and replacing the default sequence representation with pretrained Sei‐derived regulatory features further improved cross‐region prediction. In cross‐sample prediction, although XChrom was influenced by batch effects in scRNA‐seq data between training and test samples, it still achieved superior performance in most classification metrics after appropriate integration of the scRNA‐seq representations. Its performance in preserving cell identity was intermediate among Seurat, LS_Lab, and MultiVI, while consistently outperformed raw scATAC‐seq data.

Additionally, XChrom exhibits strong interpretability, facilitating understanding of both *cis*‐ and *trans*‐regulatory mechanisms at single‐cell resolution. For local interpretation, through ISM analysis, XChrom enables assessment of the contribution of individual sites within a *cis*‐regulatory element to chromatin accessibility, offering a new approach for prioritizing non‐coding genetic variants. For global interpretation, by inserting TF motif sequences into background sequences, XChrom can infer TF motif activity at the single‐cell level, providing a new perspective to study cell type‐specific *trans*‐regulatory mechanisms beyond gene expression.

These features enable us to apply XChrom to entirely new scenarios. Using a paired scRNA‐seq and scATAC‐seq dataset from the primary motor cortex of four species, we demonstrated that XChrom trained on human and mouse data achieved excellent prediction performance in other species as well. In some cases, the performance of cross‐species predictions was even comparable to that of within‐species predictions. Our local interpretability analyses of chromatin accessible regions lacking homology across species revealed that XChrom does not rely on simple sequence similarity; instead, it captures evolutionarily conserved regulatory rules, such as binding sites of key TFs. These results indicate that we can train XChrom in model organisms with abundant single‐cell data and then apply it to non‐model organisms with limited data to study their genetics and evolution.

In the case study of PBMC data from COVID‐19 patients, XChrom not only accurately predicted TF activities of a novel but related cell state that was rare in the training set, but also successfully captured the dynamic changes in TF activity during the transition from mild to severe states in cMono cells. These findings indicate that XChrom does not simply memorize shallow RNA‐to‐ATAC mappings from the training data but instead learns the underlying biological principles linking cell states to chromatin accessibility. As a result, this enables XChrom to leverage readily available scRNA‐seq data to predict epigenomic features of samples with physiological or pathological conditions distinct from those in the training set, and to explore regulatory alterations underlying these conditions.

However, it is important to note that XChrom currently has some limitations. First, the model does not explicitly account for genomic variation between samples (such as single‐nucleotide polymorphisms, copy number variations, etc.), an important source of differences in sequence regulatory activity. Second, when assessing the effects of genetic variations or TF motif insertions, we assume that the cell identity remains constant, i.e., the peak embeddings and cell embeddings are independent. However, there may be a coupling between these two embeddings. Third, although XChrom can identify the activity of key TFs, the current model architecture does not directly capture the relationships between these TFs and their target genes, making it challenging to construct complete GRNs at the single‐cell level.

Future work could extend XChrom in two directions. First, XChrom could also be extended to other single‐cell cross‐modal prediction tasks. Recent methods such as scButterfly [57] and scBOND [58] have demonstrated strong performance in predicting one molecular modality from another. However, these approaches generally do not consider the sequence features underlying the modalities. In contrast, XChrom models each genomic region through its sequence, enabling predictions for regions unobserved during training. This formulation may therefore provide a useful framework for extending cross‐modal prediction to other sequence‐associated modalities, such as single‐cell DNA methylation (scDNAm). In this context, the core architecture of the sequence module and RNA‐derived cell‐state representation could in principle be retained, with the sequence module built following dedicated scDNAm models such as scMethCraft [59] to predict regional methylation levels. Second, our ablation analyses showed that Sei‐derived regulatory features improved cross‐region prediction while preserving cell‐state structure, highlighting the importance of sequence representation quality for generalization to unseen genomic regions. Accordingly, advanced foundation or pretrained models could be incorporated to provide richer representations of both cellular states and genomic sequences, such as Geneformer [60] and scGPT [61] for cell‐state representations, and AlphaGenome [62] and Nucleotide Transformer [63] for sequence representations.

## 4 Methods

### 4.1 XChrom Architecture

XChrom employs a hybrid neural network architecture to predict chromatin accessibility probabilities across genomic regions at single‐cell resolution (Figure 1a). The model consists of two primary components:

- Genomic sequence encoding module

This module is based on a CNN architecture, inspired by the deep learning framework of scBasset [9]. The input consists of DNA sequences centered around each peak defined by scATAC‐seq, with a length of 1344 bp. After one‐hot encoding, these sequences are processed through seven convolutional blocks. Each block includes a one‐dimensional convolutional layer, a batch normalization layer, a max pooling layer, and a GELU activation function. The output dimensions at each convolutional block are as follows: Initial input (1344, 4) → ① (448, 288) → ② (224, 288) → ③ (112, 323) → ④ (56, 363) → ⑤ (28, 407) → ⑥ (14, 456) → ⑦ (7, 512). Then, the feature map passes through an additional convolutional layer with a GELU activation, resulting in a dimension of (7, 256). Finally, the data flows through a fully connected layer (input dimension 1792, output dimension 32), producing a 32‐dimensional embedding for each peak region. A dropout rate of 0.2 is used in the fully connected layer to regularize the model, randomly masking neurons to improve generalization. Additionally, sequence inputs are augmented using stochastic reverse complementation (randomly flipping strand orientation with 50% probability) and random shifts (±3 bp) as implemented in scBasset. These augmentations promote strand and positional invariance during training.

ii. Cell identity encoding module

This module extracts cell identity information from scRNA‐seq data. The initial cell embedding matrix (e.g., PCA‐processed) is layer‐normalized to eliminate variance contribution differences among principal components. The normalized matrix is then processed through two fully connected layers (with 64 and 32 neurons, respectively), using ReLU and linear activation functions, producing a 32‐dimensional cell embedding matrix. To enhance the model's robustness, a data augmentation strategy is applied: isotropic Gaussian noise (with a standard deviation controlled by the hyperparameter noise factor, default 0.01) is added exclusively during training. During evaluation, the input remains unchanged. This operation is implemented with the tensorflow.keras.backend.in_train_phase() function.

The 32‐dimensional peak embedding and cell embedding matrix are combined through matrix multiplication. The result is then passed through a Sigmoid activation function, yielding continuous chromatin accessibility probabilities in the range [0, 1] across all cells.

Notably, XChrom explicitly considers the impact of single‐cell sequencing depth during prediction. After embedding integration, a learnable sequencing depth correction term is introduced for each cell. This term is implemented through a single‐neuron linear fully connected layer, allowing the model to automatically learn and adjust for sequencing depth effects on predictions. The initial input of the correction term is the sequencing depth estimated from binarized scATAC‐seq data and normalized across cells by *Z*‐scores. When generating the sequencing depth‐normalized chromatin accessibility profiles, the default input of the correction term for all cells is set to 1.

### 4.2 Software Implementation and Computational Environment

XChrom was implemented in Python 3.8 using TensorFlow‐GPU 2.6.0 and Keras 2.6.0. Major software packages used for preprocessing and evaluation included Scanpy 1.9.5, AnnData 0.9.2, and scikit‐learn 1.0.2. Versions of additional dependencies are provided in the requirements.txt file distributed with the XChrom package in the GitHub repository. In this study, model training and benchmarking were performed on an NVIDIA GeForce RTX 3090 GPU, with CUDA 11.2 and cuDNN 8. Computational resource usage for representative datasets is summarized in Figure S3.

### 4.3 Paired scRNA‐seq and scATAC‐seq Dataset Preprocessing

We collected multiple paired scRNA‐seq and scATAC‐seq datasets for model training and testing. We performed quality control (QC) on these datasets using scanpy (v.1.9.5) [21]. Specifically, for scRNA‐seq data, we excluded genes expressed in fewer than 5% of cells; for scATAC‐seq data, we excluded peaks present in fewer than 5% of cells. For the h_gonads dataset and the cross‐sample BMMC datasets, the threshold was adjusted to 1%. Additionally, we filtered out peaks not mapped to chromosomes: for human data, peaks on chromosomes 1–22, X, and Y were retained; for mouse data, peaks on chromosomes 1–19, X, and Y were retained.

### 4.4 Training/Test Set Split

To systematically evaluate model performance, we adopted the following strategies for splitting the training and test sets across different scenarios. In the within‐sample scenario, we divided the cells and peak regions at a ratio of 90%, constructing four datasets for model training and evaluation (Figure 1b).

- Training‐validation set: composed of training cells and training peaks, with 90% of peaks used for model parameter training and 10% reserved for validation.
- Cross‐region test set: composed of training cells and testing peaks, used to assess the model's ability to generalize to unseen genomic regions.
- Cross‐cell test set: composed of testing cells and training peaks, used to evaluate the model's prediction performance on unseen cells.
- Cross‐both test set: composed of testing cells and testing peaks, used to assess the model's overall generalization ability on unseen cells and genomic regions.

In the cross‐sample or cross‐species scenario, we divided the cells and peak regions of one sample at a 90% ratio for the training‐validation set, and the remaining 10% served as the within‐sample or within‐species test set. We used another independent sample as the cross‐sample or cross‐species test set to evaluate the model's transferability to different samples or species. Both cells and genomic regions were randomly split into training and test sets using a fixed random seed of 20.

### 4.5 Model Evaluation Metrics

Since XChrom predicts binarized chromatin accessibility (with sequencing count ≥ 1 as the true label), auROC and auPRC are the primary evaluation metrics for the model's discriminative ability. While auROC is relatively insensitive to class imbalance, auPRC is a more informative metric for highly imbalanced datasets. These metrics were calculated at three levels:

- Overall metrics: calculated across all test cells and peak regions;
- Per‐peak metrics: calculated for each peak region across test cells, then averaged over all test peak regions;
- Per‐cell metrics: calculated for each cell across all test peak regions, then averaged over all test cells.

We also adopted NS and LS to evaluate the cell identity fidelity of raw and denoised scATAC‐seq data following scBasset. Both metrics were calculated using KNN graphs at different neighborhood scales (*k* = 10, 50, 100), which were constructed in PCA‐reduced space. Specifically, let $\;N_{k}^{true}\left(i\right)$ denote the set of *k*‐nearest neighbors of cell *i* in the paired scRNA‐seq space, and $N_{k}^{imp}\left(i\right)$ the corresponding set in the imputed scATAC‐seq latent space. NS quantifies the preservation of local cell‐state geometry between the two modalities by measuring the fraction of shared nearest neighbors:

$$NS_{k}=\frac{1}{N}\sum_{i=1}^{N}\frac{\left|N_{k}^{true}\left(i\right)\;\cap N_{k}^{imp}\left(i\right)\right|}{k}$$

LS instead quantifies local cell‐type consistency in the imputed scATAC‐seq space:

$$LS_{k}=\frac{1}{N}\sum_{i=1}^{N}\left(\frac{1}{k}\sum_{j\in N_{k}^{\mathrm{imp}}\left(i\right)}I\left(y_{j}=y_{i}\right)\right)$$

where *N* is the number of evaluated cells, *y*_i_ denotes the reference cell‐type label of cell *i*, and I(·) is the indicator function, which returns 1 if the specified condition is true and 0 otherwise. When evaluating on the test set, *N* denotes the number of cells in the test set, and the index *i* iterates exclusively over the test cells. Since ground‐truth cell types were unavailable for some datasets, Leiden clusters derived from scRNA‐seq data were used as proxy cell‐type labels.

We further evaluated several conventional clustering metrics in the denoised accessibility profiles using ARI, AMI, and ASW. ARI measures the agreement between two partitions while correcting the Rand index for agreement expected by chance, whereas AMI quantifies the mutual information between two partitions with adjustment for chance. For ARI and AMI, Leiden cluster assignments derived from the denoised accessibility profiles were compared with the corresponding reference cell type labels. The clustering resolution was chosen to yield the same number of clusters as in the RNA‐derived reference partition. ARI and AMI were calculated using the adjusted_rand_score and adjusted_mutual_info_score functions, respectively, from scikit‐learn (v.0.22.1). ASW evaluates the separation of cells according to their reference cell type labels. For each cell, the silhouette coefficient compares its mean distance to cells of the same type with the mean distance to the nearest other cell type. The ASW was calculated as the mean silhouette coefficient across all cells using the silhouette_score function in scikit‐learn (v.0.22.1). Following scBasset and previous single‐cell benchmarking studies [64], the cell type ASW was rescaled from its original range of ‐1 to 1 to a range of 0 to 1 as $\frac{ASW+1}{2}$, with higher values indicating better preservation and separation of cell type structure.

### 4.6 XChrom Training Strategy

Model parameters were updated using the Adam optimization algorithm, with hyperparameters set to β_1_ = 0.95 and β_2_ = 0.9995. The learning rate was decayed exponentially, starting at 0.01 and multiplied by a decay factor of 0.9 after every 10000 training steps. The loss function used was binary cross‐entropy, with binary classification accuracy, auROC, and auPRC monitored simultaneously as evaluation metrics during training. Early stopping was employed, with training terminated if the improvement in auROC on the training set was less than 1 × 10^−^^6^ over 50 consecutive training epochs. The default maximum number of training epochs was set to 1000. During training, model checkpoints were saved every 10 epochs, and NS (*k* = 100) and LS (*k* = 100) calculated from the resulting cell embeddings could be used for monitoring cell‐state preservation during training (Figure S5a).

The default number of training epochs was set based on the following empirical observations. Taking the h_PBMC dataset as an example, the training/validation loss and auROC/auPRC largely stabilized within approximately 250 epochs (Figure S1), whereas NS and LS continued to improve and approached a plateau at around 400 epochs (Figure S5b). ASW showed further improvement during intermediate training and became nearly stable by approximately 600 epochs. Between epochs 250 and 600, the mean distance to the nearest other cell type (*b*) increased more strongly than the mean within‐cell‐type distance (*a*), indicating enhanced separation between closely related cell types (Figure S5c). This trend was also evident in UMAP visualizations (Figure S5d). Thus, most additional refinement of cell type structure occurred between approximately 250 and 600 epochs, with substantially smaller improvements thereafter. Because the training duration required for convergence is expected to depend on dataset complexity, including the number and heterogeneity of cell types and states, we set 1000 epochs as a conservative default maximum. Users may adjust this setting according to the convergence behavior of their own datasets.

### 4.7 Benchmark With scBasset

scBasset [9] is a sequence‐based CNN method to model single‐cell ATAC‐seq data. We followed the guidelines on the scBasset GitHub repository at https://github.com/calico/scBasset. scBasset takes a scATAC‐seq anndata file and a genome fasta file as input. The genome fasta file can be downloaded from https://hgdownload.soe.ucsc.edu/downloads.html. We used the command‐line tool ‘scBasset/bin/scbasset_preprocess.py’ to extract sequences underlying peak regions, one‐hot encode them, and save them in sparse h5 format. The model was trained using ‘scBasset/bin/scbasset_train.py’, and model selection was based on training auROC. Default training parameters were adopted: –bottleneck 32, –batch_size 128, –lr 0.01, –epochs 1000. In the within‐sample scenario, we used the predict() function to obtain the model's predictions on the test set and calculate auROC and auPRC. We applied the imputation_Y_normalize() function to obtain the model's denoised (sequencing depth‐normalized) results, and computed the NS and LS metrics on the denoised matrix.

### 4.8 Benchmark With Seurat

To predict chromatin accessibility, we followed the guidelines on the Seurat (v.4.4.0) [5] website at https://satijalab.org/seurat/archive/v3.2/integration.html. We used the FindTransferAnchors() function with the parameter reduction = ‘cca’ to build anchors between training and testing scRNA‐seq data, choosing the first 30 canonical correlation analysis (CCA) dimensions (dims = 1:30). We then applied the TransferData() function to transfer scATAC‐seq data from training set to test set using the found anchors, with k.weight = 10 specifying the number of KNNs. In the within‐sample scenario, when visualizing the prediction results using UMAP, Seurat can only make predictions for the test data. Therefore, we used raw scATAC‐seq data to supplement and construct a complete dataset as the plot background, but the raw scATAC‐seq data were not involved in metric calculations. For each sample, we maintained the same training and test set divisions as in XChrom to ensure consistency in evaluation.

### 4.9 Benchmark With LS_Lab

We followed the tutorial provided on the neurips2021_multimodal_topmethods GitHub repository: https://github.com/openproblems‐bio/neurips2021_multimodal_topmethods/blob/main/src/predict_modality/methods/LS_lab/run/script.py/. For the “gex2atac” task, LS_Lab [6] uses a KNN regression model to predict scATAC‐seq data from scRNA‐seq data through 10‐fold cross‐validation. We first performed dimensionality reduction on both scRNA‐seq and scATAC‐seq data using the TruncatedSVD() function with n_components = 50, and then applied KNeighborsRegressor() with parameters n_neighbors = 25 and metric = ‘minkowski’. This model learned the mapping between the two modalities in the latent space. The predictions in the latent space were projected back into the original feature space by multiplying them with the component matrix from the dimensionality reduction model. The final prediction was obtained by averaging the 10‐fold cross‐validation results [4]. For each sample, we maintained the same training and test set divisions as in XChrom to ensure consistency in evaluation.

### 4.10 Benchmark With MultiVI

We followed the guidelines provided on the MultiVI (scvi‐tools v1.2.0) [7] website at https://docs.scvi‐tools.org/en/stable/tutorials/notebooks/multimodal/MultiVI_tutorial.html. We used the get_accessibility_estimates() function of MultiVI to predict chromatin accessibility from scRNA‐seq data. All parameters were kept at default settings. For each sample, we maintained the same training and test set divisions as in XChrom to ensure consistency in evaluation.

### 4.11 Benchmark With Denoising/Imputation Methods

To further evaluate the denoising performance of XChrom, we compared it with two established scATAC‐seq data enhancement methods, SCALE [15] and scCASE [17]. SCALE (v1.1.2) was applied to the complete scATAC‐seq accessibility matrix using SCALE_function with impute = True and min_peaks = 0; all other parameters were kept at their default values. The imputed accessibility matrix returned in adata.obsm[“impute”] was used for downstream evaluation. scCASE (v0.2.5) was applied using the scCASE.run function with method = “scCASE” and threshold = 0.01. Because scCASE requires the number of cell clusters *K* as an input parameter, we set *K* to the number of Leiden clusters identified from the paired scRNA‐seq data for each dataset. The enhanced accessibility matrix returned by scCASE was subsequently used for downstream evaluation. The denoised/enhanced profiles generated by SCALE and scCASE were evaluated using the same cell‐state fidelity metrics as XChrom, including NS, LS, ARI, AMI, and ASW.

### 4.12 XChrom Ablation Studies

To assess the contribution of the cell module and sequence module, we constructed a series of XChrom variants by modifying one component at a time. Unless otherwise specified, all variants were trained and evaluated using the same training/test set, optimization settings, and evaluation procedures as the default XChrom model.

#### 4.12.1 Cell Module Variants

- **PCA_no_dense**: the 32‐dimensional PCA representation derived from paired scRNA‐seq data was directly provided to the downstream integration module without the dense layers used in the default cell‐processing module;
- **PCA_larger_dense**: the default cell‐processing network (32→64→32) was replaced with a wider and deeper network (32→256→128→32), increasing cell module parameters from 4256 to 45536, while retaining the same 32‐dimensional output representation;
- **scVI**: the original 32‐dimensional PCA representation was replaced with a 32‐dimensional latent representation learned using scVI (v1.2.0) [18, 65]. scVI was initialized with n_hidden = 768, n_latent = 32, n_layers = 1, dropout_rate = 0.2, and latent_distribution = “normal”. The AnnData object was registered using SCVI.setup_anndata(), the model was trained using SCVI.train(), and the latent cell representation was extracted using SCVI.get_latent_representation();
- **cell_index**: paired scRNA‐seq‐derived cell information was replaced with non‐informative cell indices, which were passed through a trainable tf.keras.layers.Embedding layer with an embedding dimension of 32. The embeddings were randomly initialized and jointly optimized with the remaining XChrom parameters during training. Under this setting, cell identity was learned solely from the scATAC‐seq representation.

#### 4.12.2 Sequence Module Variants

- **seq_index**: the DNA sequence input was replaced with non‐informative sequence indices, which were passed through a trainable tf.keras.layers.Embedding layer with an embedding dimension of 32. These embeddings were randomly initialized and jointly optimized during model training;
- **simpler_CNN**: the default sequence encoder was replaced with a simplified encoder consisting of three convolutional layers with 128, 128, and 64 filters and kernel sizes of 15, 9, and 5, respectively, followed by a fully connected layer that projected the sequence representation to a 32‐dimensional peak embedding;
- **Sei**: the default XChrom sequence representation was replaced with pretrained Sei [19]‐derived sequence features. Peak coordinates were provided to the official Sei 1_sequence_prediction.sh workflow (https://github.com/FunctionLab/sei‐framework) using the hg38 reference genome and GPU acceleration. Sei generates predictions for 21907 chromatin profiles for each input sequence; these 21907‐dimensional regulatory profiles were subsequently projected through two dense layers (21907→1024→32) to obtain the 32‐dimensional peak representation used by XChrom.

### 4.13 Simulation Analysis of Varying B‐cell Proportions in the Training Set

To evaluate the sensitivity of XChrom to cell type abundance in the training data, we performed an analysis using the h_PBMC dataset (2714 cells). Cells were first randomly divided into training (90%) and test (10%) sets. All B cells originally assigned to the training set were then collected as a B‐cell pool (176 cells). Training sets with different B‐cell proportions (0%, 1%, 3.7%, and the original 7.3%) were then generated by sampling the corresponding number of B cells from this pool. For each condition, non‐B cells were randomly sampled from the original training pool so that the total number of training cells remained fixed (random seed = 42). The same test set was used for all conditions, and the numbers of training and test cells were fixed at 2267 and 271, respectively.

B‐cell‐specific peaks were identified from both the observed scATAC‐seq data and XChrom‐predicted accessibility profiles. For the observed scATAC‐seq data, counts were normalized to 10^4^ per cell using scanpy.pp.normalize_total() and log‐transformed using scanpy.pp.log1p(). Differential accessibility between B cells and all remaining cells was then calculated using scanpy.tl.rank_genes_groups() with the Wilcoxon rank‐sum test. Peaks with adjusted *p* < 0.05 were ranked by log‐fold change, and the top 1000 peaks were retained. XChrom predicted accessibility profiles were directly subjected to the same B‐cell‐vs.‐rest differential analysis without additional normalization. The overlap between the top 1000 B cell‐specific peaks identified from the observed and predicted accessibility profiles was quantified using the Jaccard index, $J\left(A,B\right)=\frac{\left|A\cap B\right|}{\left|A\cup B\right|}\;$.

### 4.14 Batch Effect Correction and Comparison of scRNA‐seq Integration Strategies

To evaluate the influence of scRNA‐seq preprocessing and integration on XChrom prediction, we compared four strategies: Scanpy ingest [21], Harmony [22], Scanorama [23], and scVI [18, 65]. Scanpy v1.9.5 was used throughout the scRNA‐seq preprocessing workflow. For Scanpy ingest, query and reference datasets were first restricted to their shared genes, after which query cells were mapped onto the reference PCA space using sc.tl.ingest() with the reference cell type annotation. For scVI (scvi‐tools v1.2.0), the AnnData object was registered using scvi.model.SCVI.setup_anndata() and a model with a 32‐dimensional latent space was trained using scvi.model.SCVI (n_latent = 32). The resulting latent representation was extracted using get_latent_representation() and used as the cell embedding. For Scanorama (v1.7.4), datasets were integrated using scanpy.external.pp.scanorama_integrate() with batch identity as the integration key, and the resulting “X_scanorama” representation was used as the cell embedding.

For the main XChrom analyses, batch effects in scRNA‐seq data were corrected using Harmony (v0.0.10). In the cross‐sample scenario, samples were first restricted to shared genes and concatenated. PCA was then performed using scanpy.pp.pca() with n_comps = 32. Harmony integration was subsequently applied using scanpy.external.pp.harmony_integrate() with sample identity as the batch variable. In the cross‐species scenario, we first concatenated samples within each species based on shared genes. Then, we obtained a one‐to‐one ortholog gene list between each species and human via BioMart [66], and used this list to retain orthologous genes for cross‐species data integration. After correction, data for each sample was saved as an h5ad file, with the corrected 32‐dimensional cell embedding matrix stored in.obsm[‘X_pca_harmony’], which was used as the initial cell embedding matrix for model training and prediction.

### 4.15 Identification of Cell‐Type‐Specific Motifs Using ISM

For the human‐specific chromatin accessibility regions (lacking homology to the mouse genome) near the *PSEN1* and *DNAJC5* genes, we performed ISM to compute single‐nucleotide importance scores at single‐cell resolution. The process was as follows: For each target sequence position, we performed three forward passes using the mouse‐trained XChrom model, mutating each reference nucleotide to one of the three alternative nucleotides while keeping the initial human‐derived cell embeddings unchanged as the model input. By comparing the chromatin accessibility predictions for each mutated sequence with the reference sequence, we computed the effect of each mutation in each cell. The ISM scores for the four nucleotides at each position were normalized such that their sum was zero, and the normalized ISM score for the reference nucleotide was used as the importance score for that position. This process yielded a complete ISM score matrix for each target peak region, with dimensions (cell number × 1344 bp × 4 nucleotides), quantifying the impact of nucleotide mutations at each position on the predicted values for each cell. For further analysis, we performed *Z*‐score normalization of the ISM scores across different cells to enable comparison of the relative importance of each nucleotide position across cell types. Additionally, we used the FIMO (v5.4.1) tool [31] to scan the target peak regions for TF motifs, setting a candidate motif matching *p*‐value threshold to 1 × 10^−^^3^. For each identified candidate motif, we computed the dot product between its position weight matrix (PWM) and the corresponding ISM score for the motif region. This yielded a specificity score for each cell. We also performed a two‐sided Wilcoxon rank‐sum test to evaluate statistical differences between the specificity scores of any two cell types.

### 4.16 Calculate TF Motif Activity

To quantitatively assess TF regulatory activity at the single‐cell level, we adopted a motif insertion‐based perturbation strategy and used the XChrom model to calculate motif activity scores for each TF in every cell. First, we applied the fasta_dinucleotide_shuffle tool [67] to randomly select and shuffle the sequences of 1000 peak regions from the training/test peak sets, generating 1000 background genome sequences as controls. In the cross‐species analysis, the background sequences were derived from peak regions in the test set; for the COVID‐19 analysis, the background sequences were constructed using the peak regions from the training set (PBMC10k).

For each TF in the motif database, we sampled 1000 representative motif sequences from its PWM, each of which was inserted into the central position of a corresponding background sequence, resulting in 1000 motif‐inserted sequences for each TF motif. We then input the motif‐inserted sequences and the original background sequences into the trained XChrom model for forward propagation, using initial cell embeddings derived from the scRNA‐seq data of the test set as input, and predicted sequencing depth‐normalized chromatin accessibility values for all cells. The difference in prediction between the motif‐inserted sequences and the background sequences was defined as the motif activity score in each cell. For each TF, we calculated the mean motif activity score across all 1000 sequences in each cell, obtaining the raw TF activity score for that cell. To visualize the activity distribution of a specific TF across different cells, we performed *Z*‐score normalization on the raw TF activity scores for all cells to facilitate comparison.

We used CIS‐BP [68] as the motif database in this analysis, which was downloaded from https://meme‐suite.org/meme/meme‐software/Databases/motifs/motif_databases.12.27.tgz. For human samples as the test set, we used the human motif database (motif_databases/CIS‐BP_2.00/Homo_sapiens.meme), and for mouse samples as the test set, we used the mouse motif database (motif_databases/CIS‐BP_2.00/Mus_musculus.meme).

### 4.17 TF Activity Correlation Between Species Across Cell Types

To assess the conservation of TF regulatory activity between species, we calculated the Pearson correlations of TF motif activity across cell types under three different settings:

- Correlation analysis using a human‐trained model: We used the XChrom model trained on human data to calculate TF motif activity in both human (m1d1) and mouse (mop3c2) samples, and then calculated the Pearson correlation coefficient of each TF's activity between human and mouse samples across cell types.
- Correlation analysis using a mouse‐trained model: We used the XChrom model trained on mouse data to calculate TF motif activity in both human (m1d1) and mouse (mop3c2) samples, and then calculated the Pearson correlation coefficient of each TF's activity between human and mouse samples across cell types.
- Correlation analysis between models from different species: We calculated TF motif activity using the human‐trained model in the human sample (m1d1) and the mouse‐trained model in the mouse sample (mop3c2), and analyzed the correlation across cell types between the two species.

For each cell type, we computed the mean activity value across all cells. All analyses were based on 677 common motifs shared between the human and mouse motif databases to ensure consistency in the comparison.

### 4.18 Annotation of PBMC10k Training Data

We used the reference mapping method from the Seurat (v4.4.0) package [5] (https://satijalab.org/seurat/articles/multimodal_reference_mapping#intro‐seurat‐v4‐reference‐mapping) to annotate cell types in the PBMC10k scRNA‐seq data. First, we loaded an annotated multimodal PBMC dataset (pbmc_multimodal.h5seurat from https://atlas.fredhutch.org/nygc/multimodal‐pbmc/) as a reference. After performing SCTransform() normalization on the query data (PBMC10k), we used the FindTransferAnchors() function (parameters: normalization.method = “SCT”, reference.reduction = “spca”, dims = 1:50) to establish cell correspondences between the reference and query data. Then, we applied the MapQuery() function (parameters: reference.reduction = “spca”, reduction.model = “wnn.umap”) to transfer cell type labels from the reference (including celltype.l1 and celltype.l2 labels) to the query data, followed by UMAP visualization using the reference's “ref.umap” coordinates. Finally, the annotated query data was saved in both h5Seurat and h5ad formats for further analysis.

Following initial annotation, we further refined monocyte subcluster annotations within PBMC10k. Using the processed COVID‐19 scRNA‐seq dataset (COVID19_MHH50/scRNA/Monocytes_scRNAseq.rds, including PCA reduction and UMAP visualization results) as a reference, we implemented a PCA‐based reference mapping method for cell type annotation. Specifically, after extracting monocytes from the PBMC10k dataset, we normalized the data using LogNormalize(), identified the top 1000 highly variable genes, and scaled the data. We then used the FindTransferAnchors() function (parameters: reference.reduction = “pca”, dims = 1:20) to establish transcriptomic similarity between the reference and query monocyte data. Finally, we mapped the reference's cell type labels (new.id: R1‐R8) to the query dataset with the MapQuery() function (parameters: reference.reduction = “pca”, reduction.model = “umap”) and performed projection using the reference's UMAP model.

### 4.19 Differential Analysis of TF Activity in the COVID‐19 Dataset

Using an XChrom model trained on PBMC10k, we predicted the TF activities for cells in the COVID‐19 scRNA‐seq dataset. Specifically, we extracted monocytes from the COVID‐19 TF activity matrix and calculated the mean differences in TF activity between R1 (*N* = 4271 cells) and other subclusters (*N* = 34178 cells). We used the two‐sided Wilcoxon rank‐sum test, with *p*‐value adjustment via the false discovery rate (FDR) correction procedure (Benjamini‐Hochberg method). The same analysis was performed for R4 (*N* = 7100 cells) vs. other subclusters (*N* = 31349 cells).

Furthermore, we analyzed the mean differences in TF activity among cMono between the severe group (*N* = 18463 cells) and the mild group (*N* = 18947 cells). Inter‐group comparisons were performed using the two‐sided Wilcoxon rank‐sum test, with *p*‐value adjustment via FDR correction (Benjamini‐Hochberg method). We visualized the results using a volcano plot (Figure 7d), with mean differences on the *x*‐axis and adjusted *p*‐values on the *y*‐axis. The *x*‐axis was scaled using a pseudo‐log transformation from the scales package (scale_x_continuous(trans = scales::pseudo_log_trans(base = 3, sigma = 0.001), breaks = scales::pretty_breaks(n = 3))). Additionally, we extracted the expression matrix of cMono from the raw scRNA‐seq data and normalized it using scanpy.pp.normalize_total() and scanpy.pp.log1p() [21]. We calculated the Pearson correlation coefficients between activity and expression values across cells for 863 common TFs, with *p*‐values adjusted for multiple comparisons using FDR correction (Benjamini‐Hochberg method).

### 4.20 Motif Enrichment Analysis in COVID‐19 scATAC‐seq Data

We used the processed COVID‐19 scATAC‐seq dataset for further analysis. For monocyte subclusters (C1‐C6), we used the getMarkerFeatures() function from the ArchR (v1.0.2) [69] tool (parameters: useMatrix = “PeakMatrix”, groupBy = “Clusters”, bias = c(“TSSEnrichment”, “log10(nFrags)”), testMethod = “wilcoxon”) to identify marker peaks for each subcluster. We then applied the peakAnnoEnrichment() function (parameters: peakAnnotation = “Motif”, cutOff = “Pval ≤ 0.05 & Log2FC > 1”) to identify significantly enriched TF motifs in these marker peaks. Motif annotations were based on the CIS‐BP database [68]. According to the original study, the C1 and C4 subclusters in the scATAC‐seq data were mapped to the R1 and R4 subclusters in the scRNA‐seq data, respectively [39].

### 4.21 Statistical Analysis

For paired comparisons, the normality of the paired differences was assessed using the Shapiro‐Wilk test. A two‐sided paired *t*‐test was used when the normality assumption was satisfied; otherwise, a two‐sided Wilcoxon signed‐rank test was applied. For comparisons of PWM–ISM scores between cell types and differential TF activity analyses, two‐sided Wilcoxon rank‐sum tests were used. For analyses involving multiple comparisons, *p*‐values were adjusted using the Benjamini‐Hochberg procedure. Statistical significance was defined as *p* < 0.05.

## Author Contributions

Y.M. and Z.W. designed the study. Y.M. performed data analysis. Y.M. built the software. X.L. and W.Z. tested the software. Y.M. wrote the manuscript. Z.W., X.L., D.H., Y.L., and W.Z. revised the manuscript. Z.W. supervised the study.

## Conflicts of Interest

The authors declare no conflicts of interest.

## Supporting information




**Supporting File 1**: advs78456‐sup‐0001‐SuppMat.docx.

**Supporting File 2**: advs78456‐sup‐0002‐SuppMat.xlsx.

## Data Availability

In the within‐sample scenario, we downloaded the 10x Multiome datasets from 10x Genomics: human PBMC dataset (h_PBMC) https://www.10xgenomics.com/datasets/pbmc‐from‐a‐healthy‐donor‐granulocytes‐removed‐through‐cell‐sorting‐3‐k‐1‐standard‐1‐0‐0, mouse brain dataset (m_brain) https://www.10xgenomics.com/datasets/fresh‐embryonic‐e‐18‐mouse‐brain‐5‐k‐1‐standard‐2‐0‐0, and human healthy brain dataset (h_brain) https://www.10xgenomics.com/datasets/frozen‐human‐healthy‐brain‐tissue‐3‐k‐1‐standard‐2‐0‐0. The 8.6‐week female gonad dataset (h_gonads) is available from ArrayExpress (https://www.ebi.ac.uk/biostudies/arrayexpress), with accession number E‐MTAB‐11708 and sample name HCA_F_GON10535495 [70]. The mouse secondary palate at embryonic day 12.5 dataset (m_palates) is available from Gene Expression Omnibus (GEO, https://www.ncbi.nlm.nih.gov/geo/) under accession code GSE218576 [71]. In the cross‐sample scenario, we used human BMMC datasets from GEO under accession code GSE194122 [20]. In the cross‐species scenario, the 10x Multiome datasets for the motor cortex of human, mouse, macaque, and marmoset are available from GEO under accession code GSE229169 [26]. We downloaded the COVID‐19 datasets [39] from https://nubes.helmholtz‐berlin.de/s/wqg6tmX4fW7pci5/download, and PBMC10k dataset from https://cf.10xgenomics.com/samples/cell‐arc/2.0.0/pbmc_granulocyte_sorted_10k/pbmc_granulocyte_sorted_10k_filtered_feature_bc_matrix.h5. In this study, processed data after batch effect correction and ready for model training have been uploaded to Zenodo: https://doi.org/10.5281/zenodo.16959681. XChrom is freely available as an open‐source Python package at https://github.com/Miaoyuanyuan777/XChrom. Original code and scripts used for the analysis are available at https://github.com/Miaoyuanyuan777/XChrom_analysis. Tutorials are provided at https://xchrom.readthedocs.io/en/latest/index.html, covering XChrom's installation, training, evaluation, and interpretation under various scenarios described in this study, as well as its application to new scRNA‐seq samples with a pretrained XChrom model.
