# Supplementary material for: XChrom: A Cross‐Cell Chromatin Accessibility Prediction Model Integrating Genomic Sequence and Cellular Context
Source: Adv Sci (Weinh). 2026 Oct 28:e78456. Online ahead of print. doi: 10.1002/advs.78456 (PMC13715924; doi:10.1002/advs.78456)
Supplement: Supplementary file 1 [file ADVS-9999-e78456-s002.docx]

Supplementary Materials for

**XChrom: a cross-cell chromatin accessibility prediction model integrating genomic sequence and cellular context**

*Yuanyuan Miao et al.*

Corresponding author: Zhen Wang. Email: [zwang01@ucas.ac.cn](mailto:zwang01@ucas.ac.cn)

**This PDF file includes:**

Figure S1 to S18

**Other Supplementary Material for this manuscript includes the following:**

Table S1 to S10

**Supplementary Figures**

**Figure S1 Training monitoring curves across datasets in the within-sample scenario.** For each dataset, the left panel shows training and validation loss per epoch; the right panel shows training and validation auROC and auPRC per epoch. h_PBMC (a); m_brain (b); h_brain (c); h_gonads (d); m_palates (e).

**Figure S2 Cross-region prediction performance of XChrom and scBasset on a single sample.** Rows correspond to datasets (top to bottom is h_PBMC, m_brain, h_brain, h_gonads, m_palates); columns to models (left panel: XChrom, right panel: scBasset). Within each panel, the four subplots (left to right) show: (i) auROC per cell vs. log-transformed peak counts per cell; (ii) auPRC per cell vs. log-transformed peak counts per cell; (iii) auROC per peak vs. log-transformed cell counts per peak; and (iv) auPRC per peak vs. log-transformed cell counts per peak.

**Figure S3 Computational resource requirements of XChrom and scBasset.** Training time, peak GPU memory usage, and peak CPU memory usage were measured for XChrom and scBasset on the h_PBMC and PBMC10k datasets. h_PBMC contained 2,443 cells and 24,436 peaks, whereas PBMC10k contained 10,709 cells and 91,070 peaks. Both models were trained for 1,000 epochs under the same hardware settings using an NVIDIA GeForce RTX 3090 GPU. Values above the bars indicate the measured runtime or peak memory usage.

**Figure S4 Cell type-specific cross-region prediction performance of XChrom and scBasset in the h_PBMC dataset.**

a, Distribution of Shannon entropy across all peaks (*n* = 27,150), with lower entropy indicating higher cell type-specificity.

b, auPRC for XChrom and scBasset across entropy-stratified peaks. The analysis was performed on 2,714 held-out test peaks from the cross-region prediction task (entropy < 2, *n* = 58; 2 < entropy < 2.5, *n* = 274; 2.5 < entropy < 3, *n* = 639; entropy > 3, *n* = 1743).

c, Kernel density estimate (KDE) distribution of XChrom-predicted accessibility scores for observed inaccessible (*y* = 0) and accessible (*y* = 1) states. Plots compare highly cell type-specific (entropy < 2) and broadly accessible (entropy > 3) peak groups. The Kolmogorov–Smirnov (KS) statistic (*D*) between the two distributions is indicated in each panel, with larger *D* values representing stronger separation.

d, Decomposition of the training loss across epochs for different entropy-defined peak groups. Panels (left to right) display the mean loss over all observations (*L*), observed inaccessible states (*L*_0_, *y* = 0), and observed accessible states (*L*_1_, *y* = 1).

e, Spearman correlation between the XChrom-predicted accessibility and expression of HOMER-annotated genes of the corresponding highly cell type-specific peaks (entropy < 2) among the cross-region held-out test set.

f, UMAP visualization of a representative peak (chr19:43,692,339-43,694,037), annotated to *PLAUR*. Cells are colored by *PLAUR* expression, observed ATAC accessibility, XChrom-predicted accessibility, and scBasset-predicted accessibility.

**Figure S5 Characterization of XChrom training dynamics.**

a, Pearson correlation between performance metrics (NS and LS, *k*=100) computed from the final cell embeddings and the model-denoised scATAC-seq profiles. Results are shown for the h_PBMC (left panels) and m_brain (right panels) datasets.

b, Trajectories of NS (*k* = 100) and LS (*k* = 100) metrics derived from cell embeddings across five datasets over the training epochs.

c, ASW, mean intra-cluster distance (*a*), and mean distance to the nearest other cluster (*b*), calculated from the XChrom-denoised training data at different epochs in the h_PBMC dataset.

d, UMAP visualization of XChrom-denoised training data at epoch 50, 250, 600 and 1000 for the h_PBMC dataset.

**Figure S6** UMAP visualizations of raw scATAC-seq data and of the whole dataset denoised by XChrom and scBasset. Panels: m_brain (a); h_brain (b); h_gonads (c); m_palates (d).

**Figure S7 Quantitative evaluation of scATAC-seq denoising performance.** NS and LS (calculated with *k* = 10, 50, and 100 neighbors), ARI, AMI, and ASW metrics were computed for the raw scATAC-seq data (raw_ATAC) and the denoised results from SCALE, scCASE, and XChrom. Statistical significance was assessed using two-sided paired tests (Table S4). *N*=5 datasets. Only significant comparisons are indicated: **P* < 0.05; ** *P* < 0.01; *** *P* < 0.001.

**Figure S8** UMAP visualizations of test cell predictions by XChrom, Seurat, LS_Lab, and MultiVI across datasets. m_brain (a); h_brain (b); h_gonads (c); m_palates (d). Larger points denote test cells, while background points denote training cells.

**Figure S9 Evaluation of XChrom prediction sensitivity across varying model components and cell type abundances in the h_PBMC dataset.**

a, Ablation study of the cell module. Model variants include: (i) PCA_no_dense (direct integration of 32-dimensional paired scRNA-seq PCA vectors with the sequence module without dense layers); (ii) PCA_larger_dense (replace the default cell-processing network with a higher-capacity neural network, cell module parameters from 4,256 to 45,536); (iii) scVI (use scVI-derived cell embeddings instead of PCA-derived embeddings); and (iv) cell_index (paired scRNA-seq-derived cell information was replaced with non-informative cell indices). XChrom represents the default configuration.

b, UMAP visualizations illustrating the denoising performance of the cell_index and simpler_CNN ablated models. Test cells are highlighted as larger, darker points.

c, Ablation study of the sequence module. Model variants include: (i) seq_index (DNA-sequence input was replaced with non-informative sequence indices and prediction relied only on the cell-context branch); (ii) simpler_CNN (a simplified sequence encoder with three convolutional neural network layers); (iii) Sei (utilize Sei-derived sequence features to replace the default sequence encoder). XChrom represents the default configuration.

d, Jaccard index between B cell marker peaks identified from predicted accessibility scores by models trained with different B cell proportions (0, 1%, 3.7%, and 7.3% (the original proportion in the h_PBMC dataset)) and the ground-truth marker peaks identified from raw scATAC-seq data.

e, UMAP visualizations of the test cells across different training B cell proportions. B cells are emphasized as larger, dark blue points.

**Figure S10** **Impact of batch correction strategies on XChrom cross-sample prediction performance.** Quantitative evaluation metrics (auROC, auPRC, NS, and LS) were calculated by training XChrom on sample s1d1 (training set) and evaluating it on sample s2d1 (test set). To assess the influence of preprocessing on model efficacy, initial cell embeddings for XChrom cell module were generated using various integration methods prior to training, including: no-correct (raw PCA-processed cell embeddings without integration), ingest, Harmony, Scanorama, and scVI.

**Figure S11 Batch-effect correction in the cross-sample scenario with Harmony.** UMAP visualizations of the training and test sets before and after Harmony-based batch correction (treating each sample as a batch). For each panel, columns show before Harmony correction (left) and after Harmony correction (right). Panel titles label the ‘training set—test set’ sample pair. Panels: s1d1-s1d2 (a); s1d1-s2d1 (b); s2d5-s3d10 (c); s3d3-s4d1 (d); s2d4-s4d8 (e).

**Figure S12 Cross-sample (one-to-one) prediction.** UMAP visualizations of raw scATAC-seq and predictions from XChrom, Seurat, LS_Lab, and MultiVI for each training–test sample pair. Columns (left to right): raw_ATAC, XChrom, Seurat, LS_Lab, MultiVI. Panel titles indicate the training–test sample pair. Panels: s1d1-s1d2 (a); s1d1-s2d1 (b); s2d5-s3d10 (c); s3d3-s4d1 (d); s2d4-s4d8 (e).

**Figure S13** **Batch-effect correction in the cross-species scenario with Harmony.** UMAP visualizations of the different species samples before and after Harmony-based batch correction (treating each sample as a batch). Before batch correction (a) and after batch correction (b). For each panel, left to right, colored with species, samples, and cell types.

**Figure S14** **XChrom’s prediction performance in the cross-species scenario.**

a, UMAP visualizations of predictions generated by the mouse_model when applied to the test sets from four different species. Sample names are labelled in the plot.

b, Comparison of model performance between human_model and mouse_model using per cell/peak auROC (left) and per cell/peak auPRC (right) values computed from predictions on all samples. Statistical significance was assessed using two-sided paired tests (Table S7). For direct comparisons between the two models, sample sizes were *N* = 4 (human), *N* = 7 (mouse), *N* = 3 (macaque), and *N* = 4 (marmoset). Only significant comparisons are indicated: **P* < 0.05; ** *P* < 0.01; *** *P* < 0.001.

c, Comparison of model performance between human_model and mouse_model using NS/LS (*k*=10) (left) and NS/LS (*k*=50) (right) values computed from predictions on all samples. Statistical significance was assessed using two-sided paired tests (Table S7). For direct comparisons between the two models, sample sizes were *N* = 4 (human), *N* = 7 (mouse), *N* = 3 (macaque), and *N* = 4 (marmoset). For baseline comparisons against raw_ATAC, sample sizes were *N* = 5 in the human datasets and *N* = 8 in the mouse datasets. Only significant comparisons are indicated: **P* < 0.05; ** *P* < 0.01; *** *P* < 0.001.

**Figure S15** **Cross-species correlations of TF-motif activity.** Pearson correlations between human and mouse across cell types, using the 677 motifs shared between the human and mouse motif databases. Panels (left to right): left, human_model predictions for both human and mouse samples; middle, mouse_model predictions for both human and mouse samples; right, human_model predictions for the human sample and mouse_model predictions for the mouse sample.

**Figure S16** **XChrom interpretability analysis using the *PSEN1* regulatory sequence in the cross-species scenario.**

a, Visualization of mouse_model predictions for a *PSEN1* regulatory sequence in the human sample (m1d1), with reference data from the WashU Comparative Epigenome Browser (https://epigenome.wustl.edu/BrainComparativeEpigenome/). The blue-shaded sequence represents the human region (chr14: 73167855-73169199) that lacks homology to the mouse genome. Subsequences matching MEF2C and ASCL1 motifs are highlighted with blue dashed boxes in the cell-type-averaged ISM score plot.

b, Per-cell PWM-ISM scores for MEF2C and ASCL1 in excitatory (including L4/5 IT, L5/6 NP, L5 ET, L6 IT, L6 IT CAR3, L6b cell types), inhibitory (including ChC, PVALB, SNCG, SST, VIP cell types), and non-neuron (including ODC, OPC, ASC, VLMC, Endo cell types). The PWM-ISM score is the dot product of the PWM and ISM scores at motif match sites (MEF2C at chr14:73168422-73168436, ASCL1 at chr14:73168385-73168397 and chr14:73168451-73168463).

c-d, UMAP visualizations of MEF2C and ASCL1 motif activity predicted by XChrom across different cell types in the human sample (top) and the mouse sample (bottom). XChrom was trained on the mouse sample (mouse_model) (c) and the human sample (human_model) (d).

**Figure S17** **XChrom interpretability analysis using the *DNAJC5* regulatory sequence in the cross-species scenario.**

a, Visualization of mouse_model predictions for a *DNAJC5* regulatory sequence in the human sample (m1d1), with reference data from the WashU Comparative Epigenome Browser (https://epigenome.wustl.edu/BrainComparativeEpigenome/). The blue-shaded sequence represents the human region (chr20: 63917325-63918669) that lacks homology to the mouse genome. Subsequences matching GLI2 and TEAD2 motifs are highlighted with blue dashed boxes in the cell-type-averaged ISM score plot.

b, Per-cell PWM-ISM scores for GLI2 and TEAD2 in excitatory (including L2/3 IT, L4/5 IT, L5/6 NP, L5 ET, L6 IT, L6 IT CAR3, L6b cell types), inhibitory (including LAMP5, ChC, PVALB, SNCG, SST, VIP cell types), and non-neuron (including MGC, ODC, OPC, ASC, VLMC, Endo cell types). The PWM-ISM score is the dot product of the PWM and ISM scores at motif match sites (GLI2 at chr20:63918019-63918033, TEAD2 at chr20: 63918041-63918053).

c-d, UMAP visualizations of GLI2 and TEAD2 motif activity predicted by XChrom across different cell types in the human sample (top) and the mouse sample (bottom). XChrom was trained on the human sample (human_model) (c) and the mouse sample (mouse_model) (d).

**Figure S18** **Application of XChrom to PBMC datasets from COVID-19 patients.**

a, UMAP visualizations of the PBMC10k and COVID-19 scRNA-seq datasets before and after Harmony-based batch correction. Rows: before correction (top), after correction (bottom). Within each panel: left subplot colored by batch; right subplot colored by cell type.

b, UMAP visualizations colored by predicted motif activity for CEBPB, TCF7, PAX5, and RUNX3 from a PBMC10k-trained XChrom model applied to the COVID-19 dataset. For visualization, activity scores for each TF motif were *Z*-score normalized across all cells. Panels (left to right): CEBPB, TCF7, PAX5, RUNX3.

**Table S1-S7**

Statistical hypothesis tests were dynamically selected based on Shapiro-Wilk normality testing of the paired differences, reporting mean differences with Cohen’s *d* for parametric data, and pseudo-medians with Wilcoxon r for non-parametric data. Detailed column descriptions and corresponding figure panels are provided as metadata headers within the data file.

Table S1 (separate file)

**Statistical comparisons of within-sample cross-region prediction performance.** The table compares the performance metrics (auROC, per cell auROC, per peak auROC, auPRC, per cell auPRC, and per peak auPRC) between XChrom and scBasset.

Table S2 (separate file)

**Statistical comparisons of within-sample cross-cell prediction performance.** The table compares the performance metrics (auROC, per cell auROC, per peak auROC, auPRC, per cell auPRC, per peak auPRC, NS, LS, ARI, AMI, and ASW) between XChrom and benchmark methods (Seurat, LS_Lab, and MultiVI).

Table S3 (separate file)

**Statistical evaluation of model generalization across different prediction tasks.**

The table compares the performance metrics (auROC, per cell auROC, per peak auROC, auPRC, per cell auPRC, and per peak auPRC) between the cross-both task (simultaneously predicting unseen regions in unseen cells) and the other tasks (cross-region and cross-cell).

Table S4 (separate file)

**Statistical comparisons of whole dataset denoising performance.** The table compares the performance metrics (NS, LS, ARI, AMI, and ASW) between XChrom and other methods (including: scBasset, scCASE, and SCALE).

Table S5 (separate file)

**Statistical comparisons in different prediction scenarios.** The table compares the performance metrics (auROC, per cell auROC, per peak auROC, auPRC, per cell auPRC, per peak auPRC, NS, and LS) of XChrom between within-sample and cross-sample scenarios.

Table S6 (separate file)

**Statistical comparisons of different methods in the cross-sample scenario.** The table compares the performance metrics (auROC, per cell auROC, per peak auROC, auPRC, per cell auPRC, per peak auPRC, NS, and LS) between XChrom and other methods (including: Seurat, LS_Lab, and MultiVI).

Table S7 (separate file)

**Statistical analysis of cross-species prediction performance.** The table presents pairwise comparisons among human_model, mouse_model, and raw scATAC-seq data (raw_ATAC). For a given species, performance metrics (auROC, per cell auROC, per peak auROC, auPRC, per cell auPRC, per peak auPRC, NS, and LS) derived from all samples within that species were pairwise compared using paired statistical tests.

Table S8 (separate file)

**Comparison of PWM-ISM dot product between cell types.** The table includes two-sided Wilcoxon rank-sum test results for the PWM-ISM dot product of TFs (MEF2C and ASCL1) in L2/3 IT, LAMP5, and MGC cell types.

Table S9 (separate file)

**Differential analysis of XChrom-predicted transcription factor (TF) activity in COVID-19 samples.** The analysis compares TF activity across specific monocyte subclusters (R1 vs. others; R4 vs. others) and evaluates differences within classical monocytes (cMono) between mild and severe COVID-19 patients. Statistical significance was assessed using a two-sided Wilcoxon rank-sum test.

Table S10 (separate file)

**Motif enrichment analysis of COVID-19 scATAC-seq subclusters.** The table presents the motif enrichment results for the C1 and C4 subclusters, computed using ArchR based on the COVID-19 independent scATAC-seq data.
